# Protococcidian *Eleutheroschizon duboscqi*, an Unusual Apicomplexan Interconnecting Gregarines and Cryptosporidia

**DOI:** 10.1371/journal.pone.0125063

**Published:** 2015-04-27

**Authors:** Andrea Valigurová, Gita G. Paskerova, Andrei Diakin, Magdaléna Kováčiková, Timur G. Simdyanov

**Affiliations:** 1 Department of Botany and Zoology, Faculty of Science, Masaryk University, Kotlářská 2, 611 37, Brno, Czech Republic; 2 Department of Invertebrate Zoology, Faculty of Biology, Saint-Petersburg State University, Universitetskaya emb. 7/9, St. Petersburg, 199034, Russian Federation; 3 Department of Invertebrate Zoology, Faculty of Biology, Lomonosov Moscow State University, Leninskiye Gory 1–12, Moscow, 119234, Russian Federation; Bernhard Nocht Institute for Tropical Medicine, GERMANY

## Abstract

This study focused on the attachment strategy, cell structure and the host-parasite interactions of the protococcidian *Eleutheroschizon duboscqi*, parasitising the polychaete *Scoloplos armiger*. The attached trophozoites and gamonts of *E*. *duboscqi* were detected at different development stages. The parasite develops epicellularly, covered by a host cell-derived, two-membrane parasitophorous sac forming a caudal tipped appendage. Staining with Evans blue suggests that this tail is protein-rich, supported by the presence of a fibrous substance in this area. Despite the ultrastructural evidence for long filaments in the tail, it stained only weakly for F-actin, while spectrin seemed to accumulate in this area. The attachment apparatus consists of lobes arranged in one (trophozoites) or two (gamonts) circles, crowned by a ring of filamentous fascicles. During trophozoite maturation, the internal space between the parasitophorous sac and parasite turns translucent, the parasite trilaminar pellicle seems to reorganise and is covered by a dense fibrous glycocalyx. The parasite surface is organised in broad folds with grooves in between. Micropores are situated at the bottom of the grooves. A layer of filaments organised in bands, underlying the folds and ending above the attachment fascicles, was detected just beneath the pellicle. Confocal microscopy, along with the application of cytoskeletal drugs (jasplakinolide, cytochalasin D, oryzalin) confirmed the presence of actin and tubulin polymerised forms in both the parasitophorous sac and the parasite, while myosin labelling was restricted to the sac. Despite positive tubulin labelling, no microtubules were detected in mature stages. The attachment strategy of *E*. *duboscqi* shares features with that of cryptosporidia and gregarines, i.e. the parasite itself conspicuously resembles an epicellularly located gregarine, while the parasitophorous sac develops in a similar manner to that in cryptosporidia. This study provides a re-evaluation of epicellular development in other apicomplexans and directly compares their niche with that of *E*. *duboscqi*.

## Introduction

Phylum Apicomplexa Levine 1980, emend. Adl et al. 2012 [1] represents one of the most successful groups of eukaryotic unicellular organisms, consisting entirely of parasitic genera that infect a broad range of vertebrates and invertebrates. In contrast to the apicomplexan etiologic agents of globally significant human and animal diseases (e.g. malaria, toxoplasmosis, cryptosporidiosis), the enormously diversified basal groups of Apicomplexa, that are restricted to invertebrate hosts, remain poorly understood. Nevertheless, they appear to be very important in the comprehension of evolutionary pathways and phylogenetic relations within the phylum Apicomplexa.

Apicomplexans evolved various adaptations for invading and surviving within their hosts. It is assumed that ancestral apicomplexans parasitised marine annelids, and their radiation and adaptation to the parasitic life style took place before the era of vertebrates. First, they spread to other marine invertebrates (turbellaria, crustaceans, echinoderms, etc.), then to freshwater and terrestrial invertebrates, and finally to vertebrates [2]. The apicomplexan zoite is characterised by a high degree of cell polarity in that it has an apical pole equipped with a so-called apical complex, usually comprising specialised secretory organelles (rhoptries, micronemes), polar rings, and a conoid. This unique invasion apparatus, traditionally used as the best defining feature for the phylum Apicomplexa, is initially linked with a myzocytosis-based mode of feeding as it is in colpodellids and archigregarines [3,4]. Most likely, apicomplexan evolution progressed from myzocytotic predation to myzocytotic extracellular parasitism, a characteristic of lower gregarines and cryptosporidia, and finally to intracellular parasitism which is typical for coccidia. This means apicomplexans demonstrate two main determinative evolutionary trends: i) the origination of intracellular parasitism in typical coccidia and Aconoidasida, accompanied by a rejection of trophozoite polarity and motility; and ii) the origination of epicellular parasitism, observed mostly in gregarines, with subsequent modifications of attachment apparatus along with the motility mode/mechanism in the trophozoite stage. Recent studies have pointed out the unique epicellular localisation of cryptosporidia within the host cell-derived parasitophorous sac (PS), and the similarities in their attachment and feeding strategy with gregarines. Thus, these parasites reflect analogous modes of adaptation to a similar environment within the host [5,6]. Based on phylogenetic analyses reporting the close affinity of gregarines and cryptosporidia [7,8], speculation that cryptosporidia represent a ‘missing link’ between the gregarines and coccidia is frequently discussed.

One of the poorly explored basal apicomplexan lineages is the order Protococcidiorida Kheisin, 1956 (subclass Coccidiasina Leuckart, 1879; class Conoidasida Levine, 1988) comprising four families: Eleutheroschizonidae Chatton & Villeneuve, 1936; Myriosporidae Grassé, 1953; Angeiocystidae Léger, 1911 and Grelliidae Levine, 1973 [9]. Protococcidia are expected to lack merogony, and their gamogony and sporogony occurs extracellularly [9]. Genus *Eleutheroschizon* Brasil, 1906 was placed in the family Eleutheroschizonidae, representatives of which are characterised by epicellular development [9,10,11]. Their gamonts detach from the host tissue and disperse into the environment where gametogenesis and sporogenesis take place. Oocysts contain fan-shaped clusters of sporozoites, with one end of each sporozoite attached to the residuum [9]. There are reported only two species of the genus *Eleutheroschizon*, the type species *E*. *duboscqi* Brasil, 1906 from *Scoloplos armiger* and *E*. *murmanicum* Awerinzew, 1908 from *Ophelia limacina* (Rathke) [11,12]. Apart from the original description [11] and one study focusing on the life cycle of *E*. *duboscqi* [10], no further studies dealing with this parasite have been published. The aim of this study was to provide a morphological analysis of the attachment strategy, cell cortex and cytoskeleton of trophozoites and gamonts *of Eleutheroschizon duboscqi*, a representative of marine apicomplexans, which shares features of both the gregarines and coccidia.

## Materials and Methods

### Material collection

The polychaetes *Scoloplos armiger* (Müller, 1776) were collected from 2006 to 2014 at the sand-silt littoral zone close to the White Sea Biological Station of M. V. Lomonosov Moscow State University (66°33.200' N, 33°6.283' E) and the Marine Biological Station of St. Petersburg State University (66°18.770' N; 33°37.715' E). Both stations are situated in the Kandalaksha Bay of the White Sea. The dissection of polychaetes and extraction of parasites were performed using a MBS-10 stereomicroscope. Squash preparations with living parasites and semi-thin sections stained with toluidine blue were investigated with the use of a Leica DM 2000 microscope connected to a DFC 420 digital camera, a Zeiss Axio Imager.A1 connected to an AxioCam MRc5 digital camera or with an Olympus microscope BX61 equipped with DP71 digital camera.

### Electron microscopy

Specimens were fixed in an ice bath in 2.5–5% (v/v) glutaraldehyde, in different concentrations of cacodylate buffer (0.05–0.15 M; pH 7.4; osmolarity was reached up to 720 mOsm by adding NaCl), for over two hours. For transmission electron microscopy (TEM), the specimens were then washed in buffer used for fixation or in filtered (0.22 μm Millipore) sea water and post-fixed in 1–2% (w/v) OsO_4_ in the same buffer for 1–3 h at room temperature. Some specimens were fixed with 3% glutaraldehyde-ruthenium red [0.15% (w/v) stock water solution] in 0.2 M cacodylate buffer (pH 7.4) and postfixed with 1% OsO_4_-ruthenium red in the same buffer. The subsequent procedure follows published protocols [6,13,14]. Observations were made using microscopes JEM-1010 (JEOL) and LEO 910 (Zeiss). For scanning electron microscopy (SEM), the specimens were washed in buffer used for fixation or in filtered (0.22 μm Millipore) sea water, processed according to Valigurová et al. [13,15] and examined using microscopes JSM-7401F —FE SEM (JEOL), LEO 420 (Zeiss) or GEMINI Supra 40V (Zeiss).

### Confocal laser scanning microscopy

Fragments of parasitised intestines were washed in 0.1 M phosphate buffered saline (PBS), fixed for 30 minutes at room temperature in freshly prepared 4% paraformaldehyde in 0.1 M PBS (PFA) or in ice-cold methanol, washed, and permeabilised for 15–30 minutes in 0.5% Triton X-100. Some of the PFA fixed samples were stained with Evans blue. Protocols for direct staining of filamentous actin (F-actin) with phalloidin—tetramethylrhodamine B isothiocyanate (phalloidin-TRITC; Sigma-Aldrich, Czech Republic), as well as indirect immunofluorescent antibody (IFA) staining using a rabbit anti-myosin antibody (smooth and skeletal, the whole antiserum), a rabbit anti-chicken spectrin antibody (the whole antiserum), a mouse monoclonal anti-α-tubulin antibody (Sigma-Aldrich, Czech Republic) and a mouse monoclonal IgG anti-actin antibody that was raised against *Dictyostelium* actin (provided by Prof. Dominique Soldati-Favre), follow Valigurová et al. [13,15]. For double labelling, specimens were incubated in phalloidin-TRITC after washing off the secondary antibody. Some preparations were counterstained with DAPI. Confocal laser scanning microscopic (CLSM) observations were made with an inverted Olympus IX81 microscope equipped with a laser-scanning FluoView 500 confocal unit (FluoView 4.3 software); using the rhodamine (Evans blue), tetramethylrhodamine isothiocyanate (TRITC—phalloidin, anti-myosin), fluorescein isothiocyanate (FITC—anti-actin, anti-α-tubulin, anti-spectrin) and/or UV (DAPI) filter sets. Some micrographs were processed using the software Fiji (an image processing package based on ImageJ developed at the National Institutes of Health). For better interpretation of fluorescent data, a set of 6 linguistic variables, such as No fluorescence (-), Very weak (-/+), Weak (+), Medium (++), Strong (+++) and Very strong (++++), were used (symbols are only illustrative and not used in the text). Quantification of fluorescence intensity was made using the visual assessment of CLSM micrographs (using the raw images), comparison was made to those obtained from two sets of control samples: negative (omitting the primary antibody or phalloidin) and positive controls (labelled, but not treated with cytoskeletal drugs).

### Experimental part

As a control for potential false positive results from the fluorescent labelling of F-actin and microtubules, specimens were treated with probes that influence the polymerisation of actin and tubulin: jasplakinolide (JAS, Invitrogen; a toxin that stabilises actin filaments and induces actin polymerisation), cytochalasin D (Invitrogen, Czech Republic; a drug disrupting actin filaments and inhibiting actin polymerisation), and oryzalin (Sigma-Aldrich, Czech Republic; a dinitroaniline herbicide acting through the disruption/depolymerisation of microtubules). Drugs were reconstituted in dimethyl sulfoxide to prepare a 1 mM stock solution. The final concentration of these membrane-permeable probes lower than 5 μM had no obvious effect. To obtain reliable results on vital cells, final solutions of 10 and 30 μM JAS, cytochalasin D and oryzalin prepared in filtered (0.22 μm Millipore) sea water were applied. Controls were performed in pure filtered sea water as well as corresponding concentrations of dimethyl sulfoxide in filtered sea water.

## Results

### Light microscopic observations

**Fig 1:**
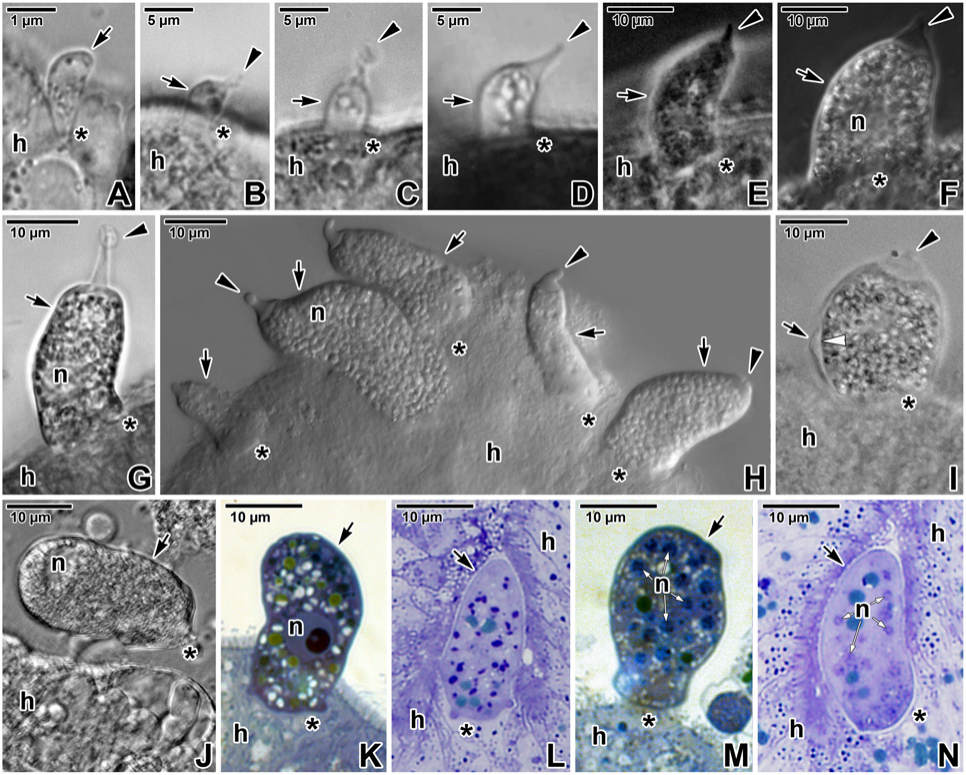
Light microscopic observations on attached stages of *Eleutheroschizon duboscqi*. **A.** Putative zoite of *E*. *duboscqi* invading the host intestinal epithelium. LM, bright field. **B-C.** Early trophozoites within an already formed PS. Note the caudal prolongation of the sac into a tail. LM, bright field. **D.** Maturing trophozoite. LM, bright field. **E.** Mature trophozoite. LM, phase contrast. **F.** A gamont stage. LM, phase contrast. **G.** Gamont exhibiting a prolonged tail at the PS. LM, bright field. **H.** Various stages of trophozoites and gamonts attached to the host intestinal epithelium. LM, differential interference contrast. **I.** A macrogamont after fixation in PFA. Note the separation of PS from the parasite cortex. LM, bright field. **J.** Detached macrogamont still enveloped by a PS. LM, bright field. **K-N.** Macrogamonts (K-L) and microgamonts (M-N) in semi-thin sections. LM, bright field, Toluidine blue. *arrow*—parasite, *arrowhead*—tail of the PS, *asterisk*—parasite attachment site, *h—*host tissue, *n*—parasite nucleus/nuclei.

We observed several development stages of *E*. *duboscqi* in squash preparations with living parasites. The earliest developmental stages, presumably zoites during the invasion process, were drop-shaped cells, 1 μm in length, with their pointed end attached to the host tissue (Fig 1A). At the light microscope (LM) level, it was not possible to identify the presence or absence of any membrane structure enclosing these parasites. The next stage was represented by helmet-shaped trophozoites, 2–10 μm in length, with a wide basal part attached to the host cell, and a large nucleus located near the base. They were enveloped by a parasitophorous sac (PS) of host membrane origin (Fig 1B–1E). The caudal part of the sac, in all attached parasites, was usually prolonged in a prominent translucent, tail-like appendage (Fig 1B–1I). The length of this tail varied. Gamonts corresponded to the helmet-shaped cells, about 20 μm in length, with a granular cytoplasm containing numerous light-refracting amylopectin granules (Fig 1F–1N). Two morphs of *E*. *duboscqi* gamonts were observed, i.e. macro- and microgamonts, agreeing with the life cycle description by Chatton and Villeneuve [10]. Macrogamonts possessed one large nucleus with a dense nucleolus (Fig 1K), while microgamonts had numerous small nuclei with fragmented nucleoli (Fig 1M and 1N). During light microscopic observations, parasites along with their PS frequently detached from host tissue (Fig 1J).

### Ultrastructural analysis

With the exception of putative zoite stages (Fig 1A), trophozoites and gamonts of various developmental stages, corresponding to our light microscopy data (Fig 1B–1N), were observed under the electron microscope. All these attached stages were covered by a PS.

**Fig 2:**
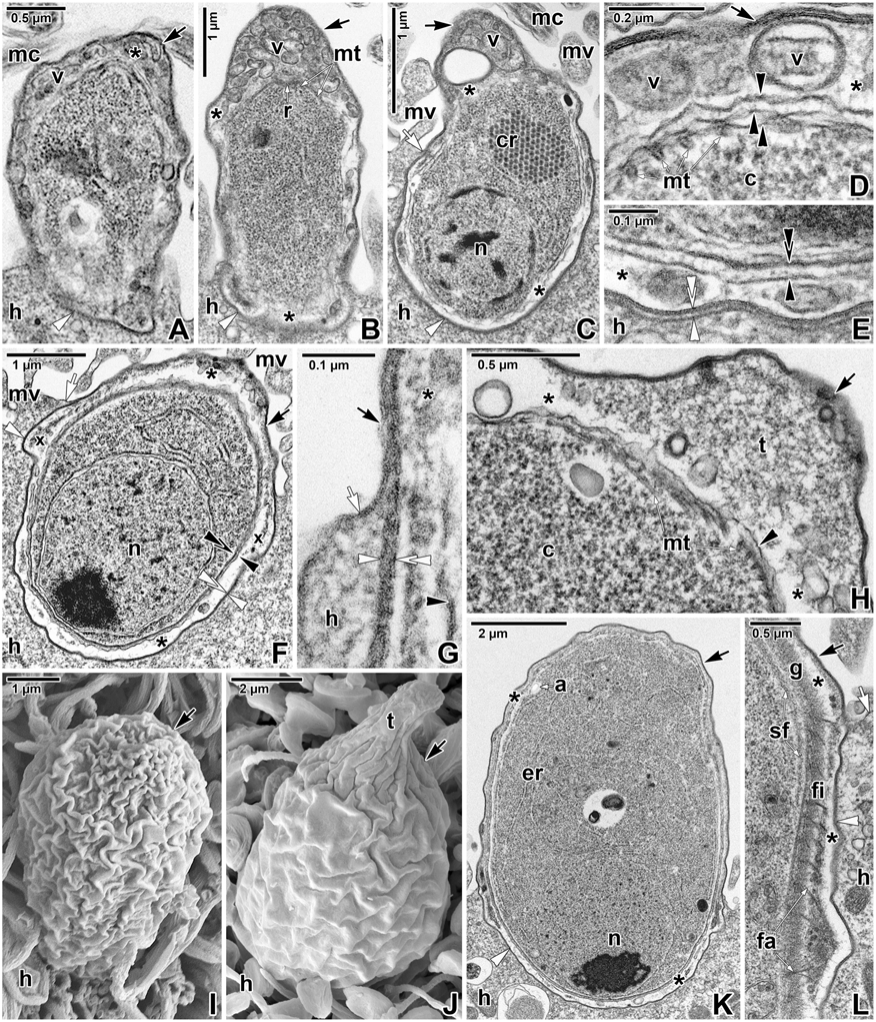
Ultrastructural features of *Eleutheroschizon duboscqi* early development. **A.** Early trophozoite in the process of transformation from an attached zoite, already enveloped by a host-derived PS. TEM. **B.** Early trophozoite. Note the numerous vesicles in the space between parasite and PS, especially in caudal region. TEM. **C-E.** Young trophozoite. D shows the space between the parasite caudal region and PS; E shows the host parasite interface at the attachment site. TEM. **F-H.** Maturing trophozoite. G shows an annular joint point of two host membranes; H focuses on the parasite caudal region and PS. TEM. **I.** Early trophozoite. SEM. **J.** Young trophozoite. SEM. **K.** Mature trophozoite. TEM. **L.** Detailed view of the attachment site of the trophozoite shown in K, focusing on the developing fascicles of filaments and the annular joint point. TEM. *a—*parasite amylopectin, *asterisk*—space between the parasite and PS, *black arrow*—PS, *black arrowhead—*parasite plasma membrane, *black double/paired arrowheads*—parasite cytomembranes, *c*—parasite cytoplasm, *cr*—crystalloid body, *er*—parasite endoplasmic reticulum, *fa*—attachment fascicles, *fi—*short attachment filaments, *g*—glycocalyx, *h—*host cell, *mc*—host microcilia, *mt*—parasite subpellicular microtubules, *mv*—host microvilli, *n*—parasite nucleus, *r*—parasite posterior ring, *sf—*parasite subpellicular filaments, *t*—tail of the PS, *v*—vesicles, *white arrow*—host cell plasma membrane, *white arrowhead*—dense band, *white double arrowhead*—base of the PS (membrane of host cell origin), *x*—forming attachment fascicles.

The earliest trophozoites, about 2 μm in length, that obviously transformed from attached zoites a short time before fixation, were already enveloped by a loose but complete PS (Fig 2A). Early trophozoites were barrel-shaped (Fig 2B). The space between the parasite and PS was filled by numerous vesicles of various sizes. The fine structure of the pellicle was not discernible in all observed early stages (Fig 2A and 2B). The attachment site at the base of PS was mostly indistinct (Fig 2B). Ultrathin sections revealed a dense, equally thick and continuous, double layer which separated the unmodified part of the host cell from its apical region bearing an attached parasite. Early trophozoites were equipped with subpellicular microtubules (21–23 nm in outer diameter) connecting to a ring-like structure, presumably a posterior ring (Fig 2B). No organelles of the apical complex were detected (Fig 2A and 2B).

**Fig 3:**
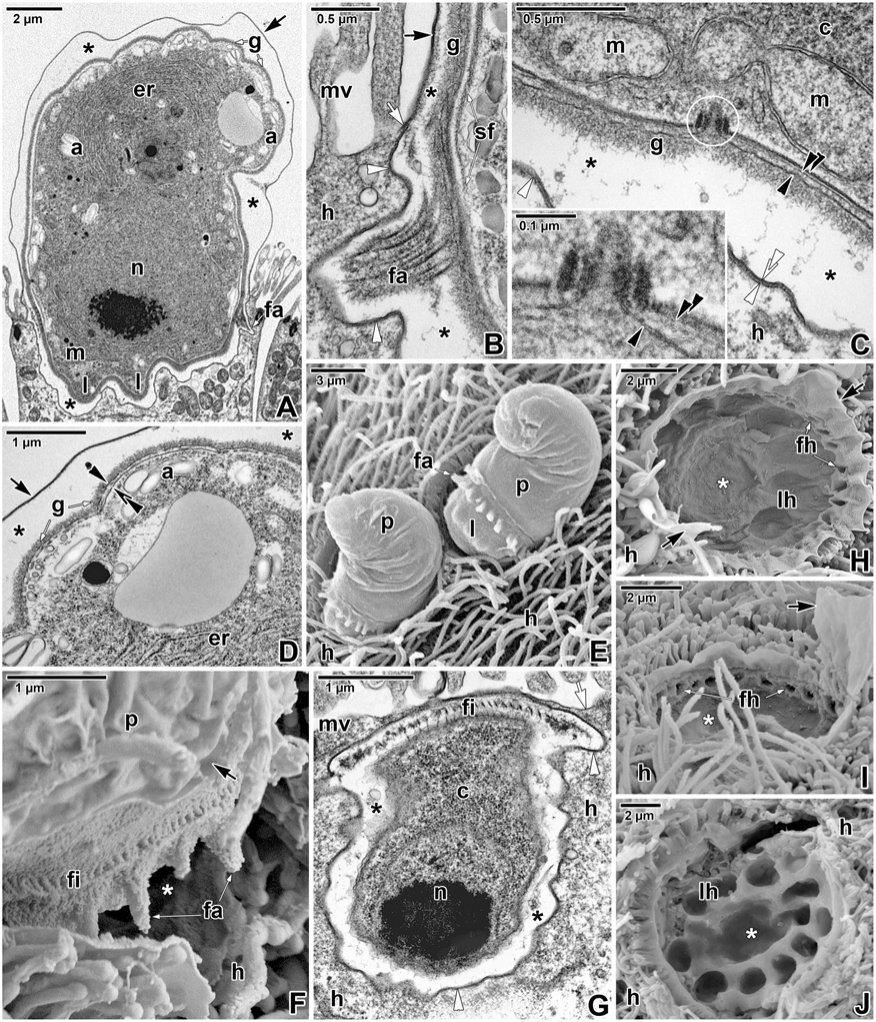
Fine structure of *Eleutheroschizon duboscqi* mature trophozoites. **A.** Mature trophozoite transforming into a gamont stage. TEM. **B.** Detailed view of the annular joint point and a well-developed fascicle of filaments. TEM. **C.** The view of mitochondria and a micropore (white circle) at the attachment site. The inset shows the micropore in detail. TEM. **D.** Higher magnification of the caudal region. TEM **E.** Two partially detached, mature trophozoites. SEM. **F.** The attachment site of a partially detached, mature trophozoite with well-developed fascicles and short filaments. SEM. **G.** Diagonal section of the apical part of a mature trophozoite. TEM. **H-I.** Craters left after detachment of mature trophozoites with well-developed attachment fascicles. Flat holes organised in one circle correspond to the developing lobes. SEM. **J.** A crater left after a trophozoite of more advanced stage as indicated by the presence of one circle of deep holes corresponding to well-developed lobes and one extra lobe starting the formation of a second circle. SEM. *a—*parasite amylopectin, *black arrow*—PS, *black arrowhead—*parasite plasma membrane, *black asterisk*—space between the parasite and PS, *black double/paired arrowheads*—parasite cytomembranes, *c*—parasite cytoplasm, *er*—parasite endoplasmic reticulum, *fa*—attachment fascicles, *fh—*holes in the host tissue left after the fascicles of the detached parasite, *fi—*short attachment filaments, *g*—glycocalyx, *h—*host cell, *l*—attachment lobe, *lh*—holes in the host tissue left after the lobes of the detached parasite, *m*—parasite mitochondria, *mv*—host microvilli, *n—*parasite nucleus, *p*—parasite, *sf—*parasite subpellicular filaments, *white arrow*—host cell plasma membrane, *white arrowhead*—dense band, *white asterisk*—empty attachment site, *white double arrowhead*—base of the PS.

During progressive maturation, the trophozoites underwent changes in their shape and size (Figs 2B–2L and 3A–3J). Gradually they took the shape of a helmet and reached up to 10 μm in length. The developmental stage, defined as a young trophozoite (3–5 μm in length), was identifiable due to a large nucleus in the apical position, a crystalloid body of unknown nature, subpellicular microtubules, and a cytoplasm rich in rough endoplasmic reticulum (Fig 2C–2E). The pellicle membranes appeared to be folded, loose and still discontinuous in some regions (Fig 2C–2E). In contrast to the earliest stages, the interface between the unmodified and modified part of the host cell at the base of the PS appeared clearly delineated, comprising of a membrane of host cell origin (about 7–9 nm in thickness, corresponding to the plasma membrane covering adjacent microvilli) underlain by a 4–7 nm thick dense band (Fig 2E). In maturing trophozoites, the next developmental stage, the pellicle seemed to be more distinct and compact along the entire surface, and its membranes became more evident than in parasites of previous developmental stages (Fig 2F). The internal space between the parasite and surrounding PS continued to clarify so that it appeared translucent with several vesicles. The border between the parasite PS and the neighbouring microvilli area formed a so called annular joint point. That is where the host cell plasma membrane, forming the base of PS, comes closer to the plasma membrane covering the neighbouring microvilli. Both membranes continuously proceed into the rising PS, forming its inner and outer membranes, respectively (Fig 2G). The 4–7 nm thick dense band underlying the inner PS membrane in the attachment site ended in this area and did not continue into the PS. No structures obviously belonging to the parasite and connected directly to the host-derived membranes of the PS were seen. In the attachment area of parasites, one ring of filaments began to form just below the annular joint point (Fig 2F). Maturing trophozoites still exhibited subpellicular microtubules (Fig 2H). In the course of trophozoite development, the caudal part of the PS gradually formed a prolongation being characteristic for *E*. *duboscqi*, the tail (Fig 2C and 2H). This is easily seen when comparing an early trophozoite without a tail (Fig 2I) with a young trophozoite with a developing tail on the PS (Fig 2J). In mature trophozoites, the next developmental stage, a cell coat (glycocalyx) appeared as a thick layer of fibrous material (Figs 2K, 2L and 3A–3D). The pellicle was distinct; it was comprised of a plasma membrane, two adjacent cytomembranes (i.e. the inner membrane complex, IMC) and a thin dense layer underlining the inner cytomembrane (Fig 3C and 3D). Under the pellicle, a thick layer of subpellicular filaments emerged (Figs 2L and 3B). The cytoplasm was filled with a large nucleus in the apical position, and an increasing rough endoplasmic reticulum. The crystalloid body observed in young trophozoites disappeared, while peripheral amylopectin granules appeared and increased in number (Figs 2K, 3A and 3D). Large mitochondria underlying the parasite pellicle appeared, especially at the attachment site (Fig 3C). With increasing age, typical apicomplexan micropores (154 nm in outer diameter and 132 nm deep when measured from the plane of cytomembranes to the bottom of the micropore) formed on the parasite surface (Fig 3C). The internal space of the PS enveloping the mature trophozoites became completely translucent (Fig 3A–3D). At the attachment site of parasite, the above-mentioned ring of filaments continued to develop: fascicles of long filaments alternating with short filaments appeared (Figs 2K, 2L, 3A, 3B and 3E–3G). The parasites formed thick outgrowths, i.e. lobes, which were organised in a single circle at the attachment site, just below the ring of filaments (Fig 3A and 3E). Correspondingly, craters in the host tissue, left after detached trophozoites, confirmed the circular organisation of the fascicles of filaments and lobes at the attachment site (Fig 3H–3J). These were seen as a peripheral circle of narrow but very deep holes, left after the well-developed fascicles of filaments, and larger flat holes, organised in one central circle and corresponding to the developing lobes (Fig 3H and 3I). In trophozoites transforming into gamonts, the attachment lobes became more and more prominent, as also documented by the deeper holes left after detached individuals (Fig 3J). In addition, the second circle of lobes started to develop in the centre of the attachment site (Fig 3A). Accordingly, detached parasites left one peripheral circle of holes corresponding to the well-developed lobes and one central, extra hole indicating the start of the formation of a second circle of lobes (Fig 3J).

**Fig 4:**
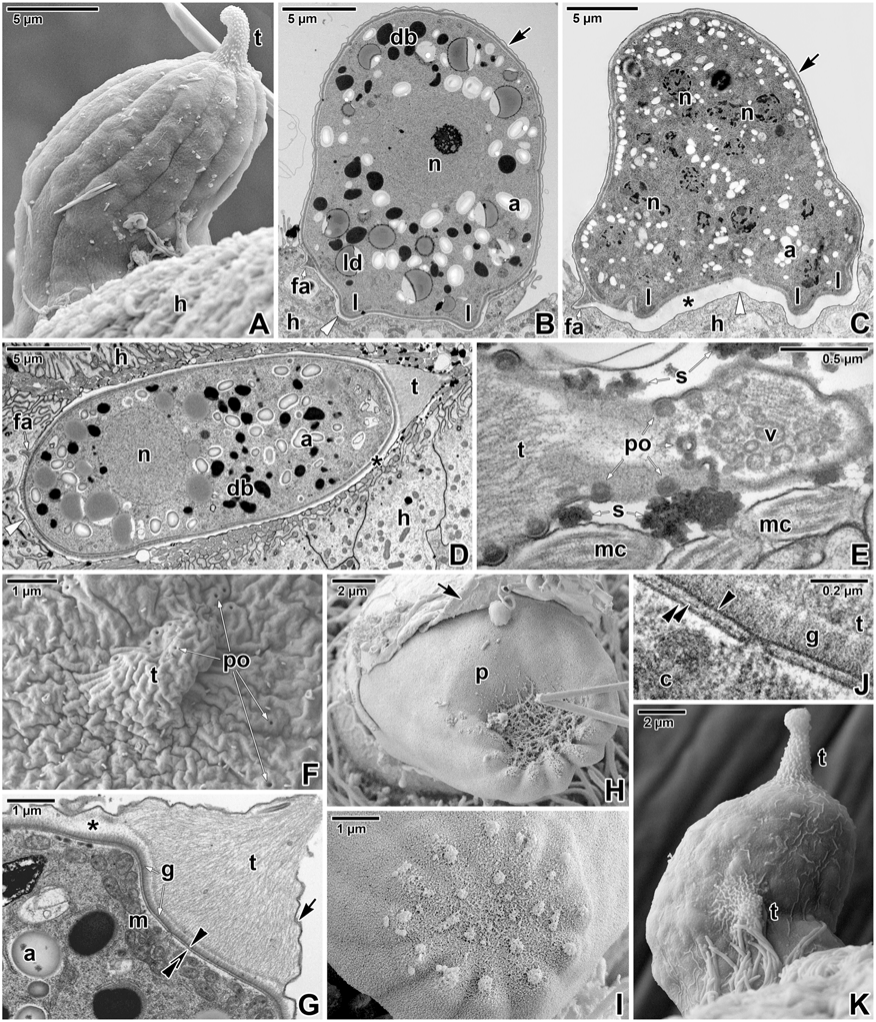
Morphology of *Eleutheroschizon duboscqi* gamonts. **A.** Attached gamont. SEM. **B.** Macrogamont with a large central nucleus. TEM. **C.** Microgamont with several nuclei. TEM. **D.** Macrogamont enclosed by host tissue. TEM, RR. **E.** The PS tail of the macrogamont shown in D. Note the pores and the mucosubstances present in their surroundings. TEM, RR. **F.** High magnification of the caudal PS part with the tail showing numerous pores. SEM. **G.** Detailed view of the tail and gamont caudal part. TEM, RR. **H.** Upper view of an individual with a ruptured PS. SEM. **I.** The caudal region of a naked individual. SEM. **J.** High magnification of the interface between the parasite and PS in the area of the tail. TEM, RR. **K.** Gamont with two tails at the PS. SEM. *a—*parasite amylopectin, *arrow*—PS, *asterisk*—space between the parasite and the PS, *black arrowhead—*parasite plasma membrane, *black double/paired arrowheads*—parasite cytomembranes, *c*—parasite cytoplasm, *db—*parasite dense bodies, *fa*—attachment fascicles, *g*—glycocalyx, *h—*host cell, *l*—attachment lobe, *ld—*parasite lipid droplets, *m*—parasite mitochondria, *mc*—host microcilia, *n—*parasite nucleus, *p*—parasite, *po*—pore, *s*—mucosubstances, *t*—tail of the PS, *v*—vesicles, *white arrowhead*—base of the PS.

**Fig 5:**
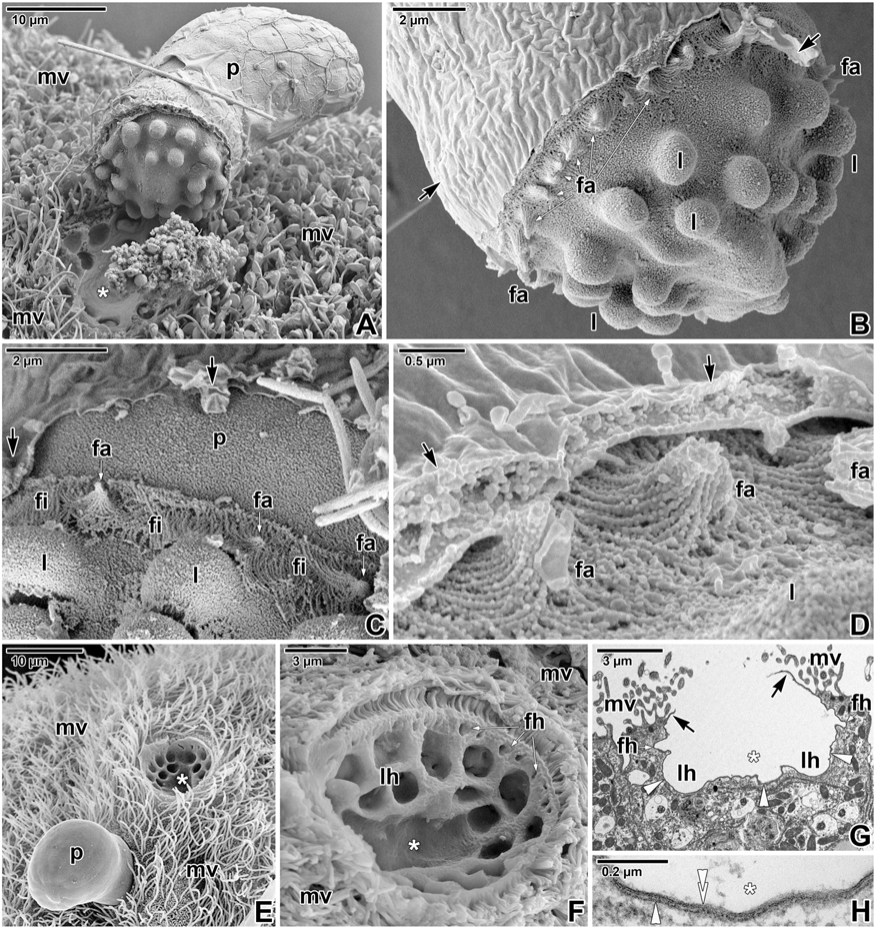
Architecture of attachment site of *Eleutheroschizon duboscqi* gamonts. **A.** Host intestinal tissue with a detached gamont revealing its attachment site at the base of PS. SEM. **B.** Detail of the gamont attachment site. SEM. **C.** Detailed view of the fascicles of long filaments alternating with short filaments, organised in ring. SEM. **D.** A detail of attachment fascicles. SEM. **E.** Host intestinal tissue with an attached parasite and a crater left after detached ones. SEM. **F.** A detail of crater left after gamont with well-developed attachment fascicles and two circles of lobes. SEM. **G.** Host epithelium showing the crater left after the parasite detached. TEM. **H.** A detail of the PS membrane remains covering the crater. TEM. *arrow*—PS, *fa*—attachment fascicle of filaments, *fh—*holes in the host tissue left after the fascicles of the detached parasite, *fi—*short attachment filaments, *l*—attachment lobe, *lh*—holes in the host tissue left after the lobes of the detached parasite, *mv*—microvilli and cilia of the host enterocyte, *p*—parasite, *white arrowhead*—dense band, *white asterisk*—empty attachment site, *white double arrowhead*—base of the PS.

**Fig 6:**
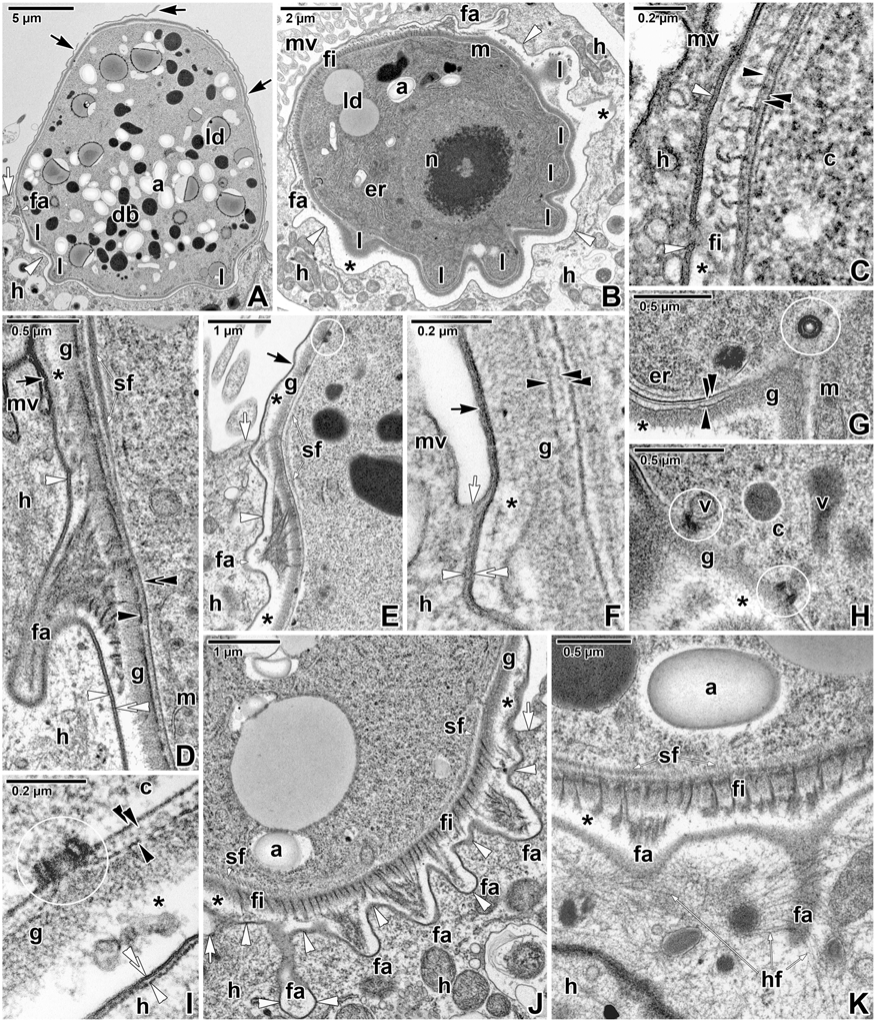
Fine structure of the attachment site of *Eleutheroschizon duboscqi* gamonts. **A.** Macrogamont with a ruptured PS. TEM. **B.** Oblique section of the attachment site. TEM. **C.** A detail showing the hook-shaped short filaments anchored into the parasite outer cytomembrane. TEM. **D-E.** The attachment fascicles in a longitudinal section. The subpellicular layer of filaments is localised just beneath the parasite IMC and ends above the fascicles. TEM; D is stained with RR. **F.** The annular joint point of two host membranes. TEM. **G.** A detail of the attachment lobe packed with endoplasmic reticulum and mitochondria. Note the cross-sectioned micropore. TEM. **H.** Detailed view of vesicles connected with the micropores located in the area of attachment lobes. TEM. **I.** Longitudinal section of a micropore localised at the parasite attachment site. TEM. **J.** Detailed view of the attachment fascicles of long filaments alternating with short filaments. TEM. **K.** The basal part of PS showing an accumulation of fine filaments in the host cell cytoplasm surrounding the PS invaginations with attachment fascicles. TEM, RR. *a—*parasite amylopectin, *asterisk*—space between the parasite and PS, *black arrow*—PS, *black arrowhead—*parasite plasma membrane, *black double/paired arrowheads*—parasite cytomembranes, *c*—parasite cytoplasm, *db—*parasite dense bodies, *er*—parasite endoplasmic reticulum, *fa*—attachment fascicle of filaments, *fi—*short attachment filaments, *g*—glycocalyx, *h—*host cell, *hf—*filaments in host cell cytoplasm, *l*—attachment lobe, *ld—*parasite lipid droplets, *m*—parasite mitochondria, *mv*—microvilli and cilia of the host enterocyte, *n—*parasite nucleus, *sf—*parasite subpellicular filaments, *v*—parasite vesicle, *white arrow*—host cell plasma membrane, *white arrowhead*—dense band, *white double arrowhead*—base of the PS. Micropores are indicated by white circles.

**Fig 7:**
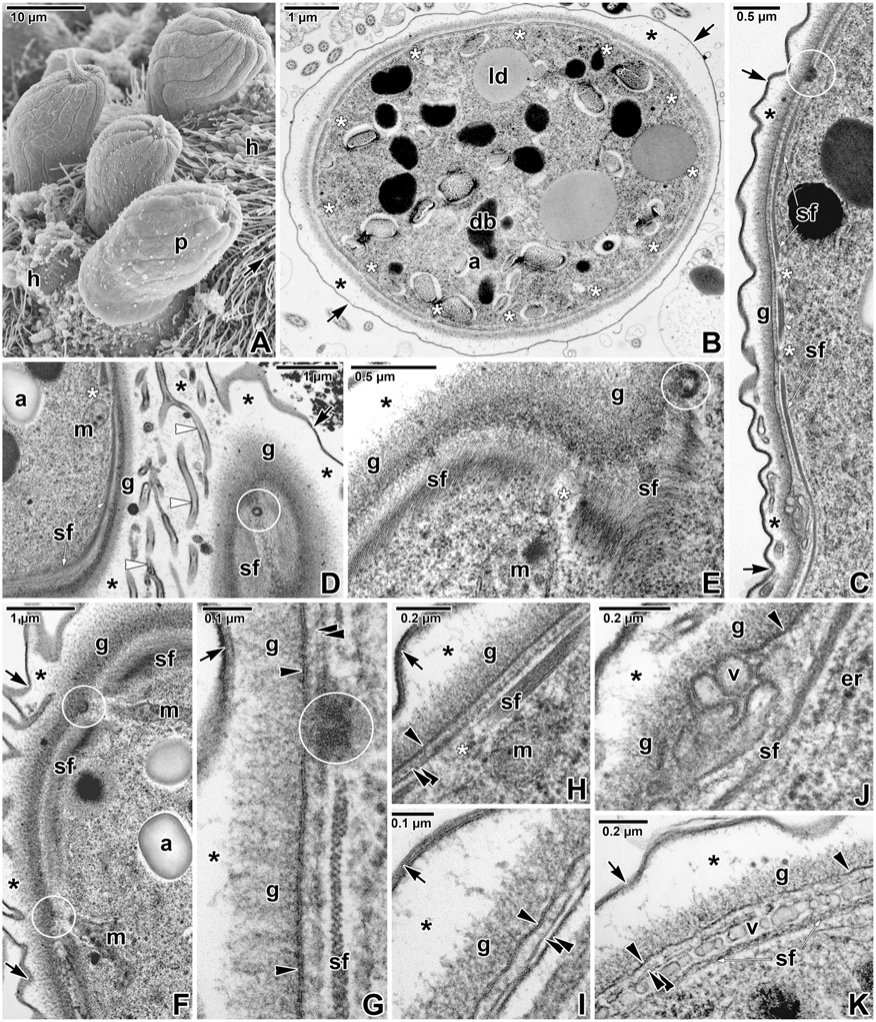
Fine structure of a parasitophorous sac and pellicle in *Eleutheroschizon duboscqi* gamonts. **A.** Attached gamonts. SEM. **B.** Cross-sectioned gamont showing its surface organised in 12 broad folds and shallow grooves corresponding to the regularly arranged interruptions of subpellicular filaments. TEM. **C.** Longitudinal section showing the organisation of PS, gamont pellicle and the subpellicular layer of filaments that is repeatedly interrupted in areas corresponding to the localisation of micropores. TEM, RR. **D.** Superficial section of the PS and the gamont pellicle. The channel-like structures located in the space between the PS and parasite correspond to the folding of the PS observed under SEM. TEM, RR. **E.** Tangential section of the gamont surface underlined with subpellicular layer of filaments. TEM, RR. **F.** Diagonal section of the gamont surface revealing mitochondria connected with micropores. TEM, RR. **G.** Cross-section of pellicle showing the subpellicular filaments interrupted in the micropore area. TEM, RR. **H.** Almost longitudinal section of pellicle with interrupted subpellicular filaments. TEM, RR. **I.** Pellicle with continuous cytomembranes. TEM. **J-K.** Re-building of the parasite IMC indicated by the discontinuous cytomembranes and numerous vesicles located between the parasite plasma membrane and the subpellicular layer of the filaments. TEM, RR. *a—*parasite amylopectin, *arrow*—PS, *asterisk*—space between the parasite and PS, *black arrowhead—*parasite plasma membrane, *black double/paired arrowheads*—parasite cytomembranes, *db—*parasite dense bodies, *er*—parasite endoplasmic reticulum, *g*—glycocalyx, *h—*host tissue, *ld*—parasite lipid droplet, *m*—parasite mitochondria, *p*—parasite, *sf—*parasite subpellicular filaments, *v—*vesicles, *white arrowheads*—channel-like structures. Micropores are indicated by white circles, interruptions of subpellicular filaments—by white asterisks.

The oldest developmental stages observed during our study were gamonts reaching about 20 μm in length (Figs 4A–4K, 5A–5E, 6A–6K, and 7A–7K). All gamonts were contained within a well-developed PS, with a prominent tail (Fig 4A and 4D–4G). A few individuals bearing two or three tails were observed (Fig 4K). The surface of gamonts exhibited about 12 (10–13, n = 20) shallow grooves showing through the PS under the SEM (Figs 4A, 4H, 4I, and 7A). Macrogamonts were characterised by a centrically located, large, roundish nucleus with one nucleolus, abundant amylopectin granules, large lipid droplets, and prominent dense bodies varying in shape and size (Fig 4B and 4D). In contrast, microgamonts possessed several nuclei (up to 20 in a longitudinal section) with fragmented nucleoli and cell inclusions (amylopectin granules, lipid droplets) of a smaller size (Fig 4C). Pores were often observed at the caudal region of the PS (Fig 4E and 4F). Dense substances staining intensively with ruthenium red (RR), most likely mucosubstances secreted by the parasite, were detected on the PS surface close to these pores (Fig 4E). The internal space between the PS and parasite was mostly translucent, while the internal space of the PS tail was packed with thin filaments running longitudinally (Fig 4E and 4G). The parasite surface (Fig 4G and 4J) bore a dense layer of fibrous glycocalyx (80–85 nm in thickness). Gamonts were often observed with a ruptured PS revealing their surface (Fig 4H and 4I). In such cases, the parasite pellicle in the caudal region was covered by a fibrous material (Fig 4H) that might correspond to the filaments observed in the PS tail under TEM. Small piles of unknown material organised in circles were observed to be present on the caudal part of the parasite surface (Fig 4I). The dense glycocalyx was visible under SEM as a woolly coat covering the surface (Fig 4I). Completely detached parasites, but still enveloped by a PS and located far away from the host tissue, were rarely detected in ultrathin sections.

Observations on mechanically detached gamonts under SEM revealed their complicated attachment sites, comprising of two circles of massive lobes crowned by a ring of fascicles of long filaments, alternating with short filaments (Fig 5A–5D). Detached individuals were covered by a PS, except for their attachment sites that seemed to be covered by a parasite pellicle only. The naked attachment lobes appeared as smooth protuberant, hemispheric structures. Detached gamonts left characteristic craters on the host intestinal tissue, indicating that they were fully matured, with well-developed attachment fascicles and two circles of lobes (Fig 5E–5G). The basal part of the PS was comprised of the host membrane (i.e. the inner membrane of PS) underlined by a dense band and was still present after parasite detachment (Fig 5H), corresponding to the observations of detached parasites with naked attachment sites.

Ultrathin sections of attached gamonts confirmed the SEM observations on the attachment site, and showed that lobes represent structures belonging to the parasite, as they are covered by a parasite pellicle and are filled with its cytoplasm packed with large mitochondria, various vesicles and the endoplasmic reticulum (Fig 6A, 6B, 6G and 6H). The pellicle membranes covering the lobes were usually well-preserved (Fig 6G). In contrast to lobes, attachment fascicles were located in the translucent space between the parasite and PS, and were not covered by the parasite pellicle (Fig 6A, 6B, 6D, 6E, 6J and 6K). Although the membranes of the parasite pellicle were not clearly distinguishable in this area, the short filaments (15–35 nm thick) and fascicles of longer filaments (about 60 nm thick) that seemed to arise from the pellicle and evidently extended through the glycocalyx, were deeply anchored into the IMC (Fig 6C–6E, 6J and 6K). Some sections clearly showed the hook-shaped short filaments that were anchored into the outer cytomembrane of IMC (Fig 6C). The subpellicular layer of filaments, localised just beneath the IMC, ended above the ring of the filaments and fascicles (Fig 6D and 6E). The organisation of structures at the annular joint point corresponded with the observations on younger stages (Fig 6F). The dense band (4–8.5 nm thick) underlying the inner PS membrane at the attachment site ended in this area. Numerous typical micropores (130–155 nm in outer diameter, 40–50 in inner diameter, the distance between the lumen of the duct and collar periphery is about 50–58 nm) were distributed at the attachment site of the parasite, especially in between individual lobes (Fig 6G–6I). Vesicles were rarely seen to be connected with micropores located at the attachment site of the parasite (Fig 6H). At the basal part of PS, an accumulation of filaments 7–10 nm thick (most likely of F-actin nature) was documented in the host cell cytoplasm surrounding the invaginations of PS with attachment fascicles (Fig 6K).

The cross-sections of mature gamonts confirmed that the surface was organised in weakly expressed broad folds with shallow grooves between them (Fig 7B). Each fold was underlain by a broad band of subpellicular filaments (apparently, it corresponds to the thick layer of subpellicular filaments observed in maturing trophozoites). Various planes of sectioning confirmed that 5–9 nm thick subpellicular filaments were oriented longitudinally (Fig 7C–7H) and formed 22–59 nm thick bands. Micropores were located at the bottom of the grooves, i.e. between these bands. Sectioning also revealed a regular arrangement of interruptions of subpellicular filaments, corresponding to the distribution of micropores (Fig 7B–7H). A lot of mitochondria could be observed in the cortical zone of the gamonts, and some were located so closely to micropores that they seemed to be connected to them (Fig 7F). In contrast to the individuals with the plasma membrane underlined by two well-preserved cytomembranes (Fig 7I), several gamonts clearly possessed discontinuous cytomembranes or a disorganised pellicle. Vesicles present in the area of subpellicular filaments can be evidence that cytomembranes underwent re-building due to pellicle reorganisation (completion or renewal) in a growing parasite (Fig 7J and 7K).

### Fluorescent visualisation of cytoskeletal elements

**Fig 8:**
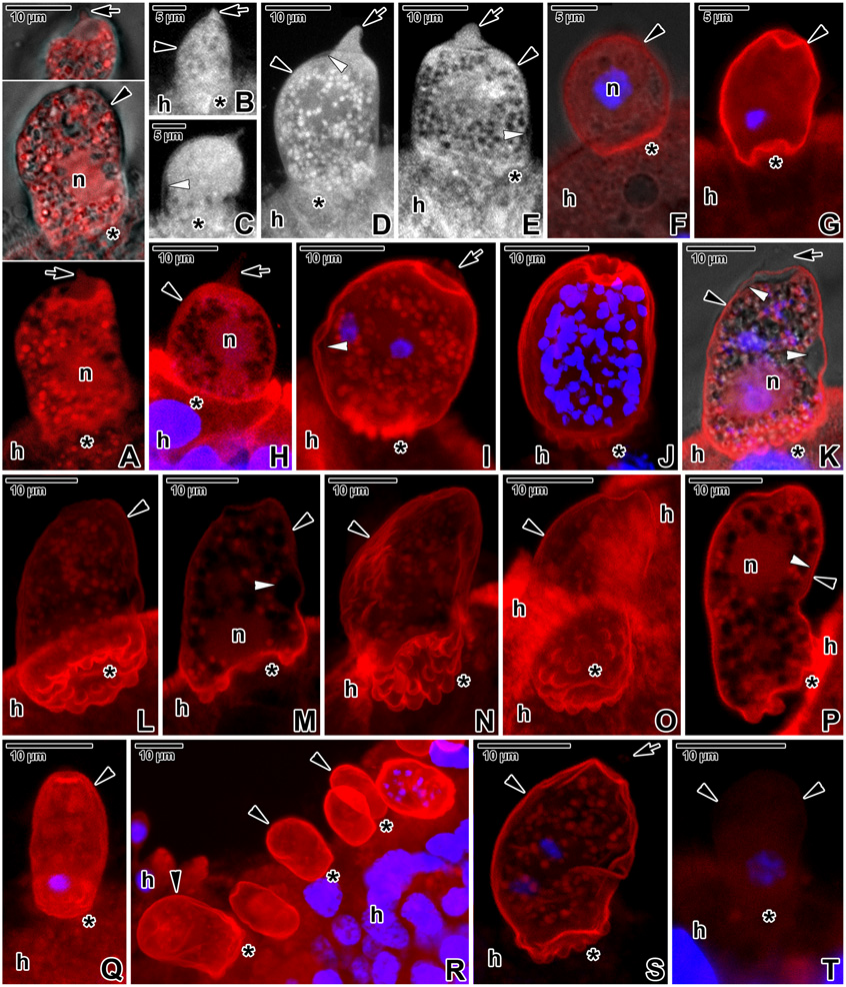
Fluorescent visualisation of an *Eleutheroschizon duboscqi* parasitophorous sac. **A.** Macrogamont stained with Evans blue. CLSM (lower) and CLSM in a combination with transmission LM (upper two). **B-E.** Trophozoites (B-D) and a gamont (E) stained with Evans blue. CLSM, output image not coloured. **F-H.** Localisation of F-actin in trophozoites. One circle of lobes is visible in the attachment site of the trophozoite shown in G. CLSM in a combination with transmission LM (F) and CLSM (G, H), phalloidin-TRITC/DAPI. **I.** F-actin labelling of a putative young microgamont with two primary nuclei. CLSM, phalloidin-TRITC/DAPI. **J.** F-actin in a microgamont with numerous nuclei. CLSM, phalloidin-TRITC/DAPI. **K-M.** F-actin in a putative macrogamont. CLSM in a combination with transmission LM (K) and CLSM (L, M), phalloidin-TRITC/DAPI. **N-P.** F-actin in a macrogamont equipped with attachment lobes organised in two circles. CLSM, phalloidin-TRITC. The intensity of signal for F-actin shown in F-P was strong for PS and medium for parasites. **Q.** Labelling of F-actin in an individual treated for 9 hours with 10 μM JAS showing the very strong labelling of PS. Individual optical sections also revealed a slightly increased F-actin labelling of the parasite. CLSM, phalloidin-TRITC/DAPI. **R.** Treatment with 30 μM JAS for 7 hours resulted in further increase of F-actin labelling in the PS, parasite and host tissue. The individual with several nuclei corresponds to the microgamont stage. CLSM, phalloidin-TRITC/DAPI. **S.** Visualisation of F-actin in an individual (putative young microgamont with two primary nuclei) treated for 9 hours with 10 μM cytochalasin D. Note the strong labelling of PS in contrast to the parasite and host tissue exhibiting only very weak signal. CLSM, phalloidin-TRITC/DAPI. **T.** Very weak F-actin labelling in a specimen treated for 7 hours with 30 μM cytochalasin D. CLSM, phalloidin-TRITC/DAPI. A-L, N-O, Q-T are composite views created by flattening a series of optical sections, while M and P represent single median optical sections. All samples were fixed in PFA. *arrow*—tail of the PS, *asterisk*—parasite attachment site, *black arrowhead*—PS, *h—*host tissue, *n—*parasite nucleus, *white arrowhead*—parasite pellicle.

For the unspecific visualisation of proteins in *E*. *duboscqi*, Evans blue staining viewed with a rhodamine filter set was used (Fig 8A–8E). Besides typical staining of cytoplasmic contents, it revealed a relatively high concentration of unspecified proteins in the PS tail and in the area of the parasite attachment site.

The strong phalloidin labelling revealed a high accumulation of filamentous actin (F-actin) in the layer corresponding to the host-derived PS as well as in the brush border of the host epithelium (Fig 8F–8P). The parasite surface and cytoplasm exhibited a fluorescent signal of medium intensity, similar to the cytoplasm of surrounding host cells. The PS tail exhibited F-actin staining of weak to medium intensity (Fig 8H and 8I). In agreement with the electron microscopic observations, the basal part of the PS enveloping the trophozoites and young gamonts showed numerous lobes organised in one circle (Fig 8F–8I), while lobes in mature gamonts formed two circles (Fig 8J–8P). The attachment site often exhibited brighter fluorescence than the rest of the parasite enclosed within the PS. After incubation in 10 μM JAS for 9 hours, the F-actin staining became very strong in the area of the PS but only slightly increased on the parasite surface (Fig 8Q). Treatment for 7 hours, with the concentration of JAS increased to 30 μM, induced even more advanced stabilisation of actin filaments, resulting in further amplification of the fluorescent signal (Fig 8R). Interestingly, the treatment with 10 μM cytochalasin D for 9 hours first caused depolymerisation of F-actin in the host tissue and parasites, while the F-actin restricted to the PS remained intact (Fig 8S) as it stained with the same intensity as non-treated controls. After incubation in 30 μM cytochalasin D for 7 hours, the fluorescence signal was very weak in all the host tissue, host-derived PS and parasites (Fig 8T).

**Fig 9:**
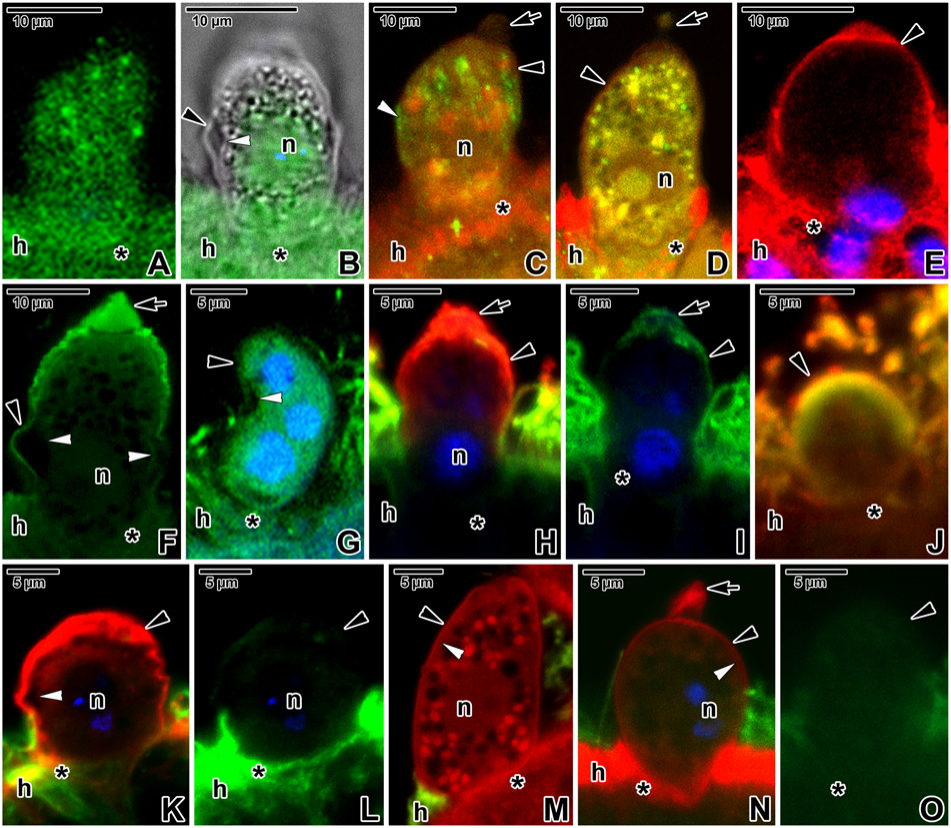
Immunolocalisation of *Eleutheroschizon duboscqi* cytoskeletal proteins. **A-B.** Actin labelling with a medium intensity in a trophozoite (PFA fixation). CLSM, IFA (A) and CLSM in a combination with transmission LM, IFA/DAPI (B). B represents a single median optical section. **C.** Actin labelling in a macrogamont treated with 30 μM JAS for 7 hours (PFA fixation). Note the increased accumulation of parasite actin (FITC) organised in longitudinal bands exhibiting strong fluorescence and strong F-actin (TRITC) labelling with a diffuse character. CLSM, IFA/phalloidin-TRITC. **D.** A gamont exhibiting a more diffuse actin (FITC) labelling of medium intensity after treatment with 10 μM cytochalasin D for 9 hours (PFA fixation). The F-actin (TRITC) labelling of the parasite did not change significantly. CLSM, IFA/phalloidin-TRITC. **E.** Very strong myosin (TRITC) labelling restricted to the PS and host tissue (PFA fixation). CLSM, IFA/DAPI. **F.** Strong spectrin (FITC) labelling of the PS in a macrogamont (PFA fixation). CLSM, IFA/DAPI. Single median optical section. **G.** Labelling of α-tubulin (FITC) of strong intensity in a young microgamont (PFA fixation). CLSM, IFA/DAPI. **H-I.** A trophozoite (fixed in ice-cold methanol) exhibiting a labelling of medium intensity for α-tubulin (FITC) and very strong intensity for myosin (TRITC). CLSM, IFA/DAPI. **J.** Labelling of α-tubulin (FITC) and myosin (TRITC) in an early trophozoite treated for 7 hours with 10 μM oryzalin (fixed in ice-cold methanol). The fluorescence signals for both antibodies did not change significantly. CLSM, IFA. **K-L.** Localisation of α-tubulin (FITC) and myosin (TRITC) in an individual (probably a young microgamont) treated with 30 μM oryzalin for 3 hours (fixed in ice-cold methanol). The fluorescence signal for tubulin became very weak, while it remained very strong for myosin. CLSM, IFA/DAPI. **M.** Co-localisation of α-tubulin (FITC) and F-actin (TRITC) in a macrogamont treated for 7 hours with 10 μM oryzalin (PFA fixation). CLSM, IFA/phalloidin-TRITC. **N-O.** Labelling of α-tubulin (FITC) and F-actin (TRITC) in a maturing trophozoite treated for 3 hours with 30 μM oryzalin (PFA fixation). CLSM, IFA/phalloidin-TRITC/DAPI. In both the preparations (M-O), there was almost no fluorescence signal for α-tubulin, while the F-actin labelled with a strong intensity. *arrow*—tail of the PS, *asterisk*—parasite attachment site, *black arrowhead*—PS, *h—*host tissue, *n—*parasite nucleus, *white arrowhead*—parasite pellicle.

Parasites labelled with the specific anti-actin antibody (known to recognise the actin in *Toxoplasma* and *Plasmodium*) exhibited fluorescence signal of medium intensity (Fig 9A and 9B). The immunolocalisation of actin differed from F-actin labelling in that the antibody did not label the PS, but labelled the host tissue with the same intensity as the parasite enclosed within the sac (Fig 9B). A slightly increased labelling was noticed at the base of the PS (Fig 9A), especially when viewing individual optical sections. A weak staining of the PS tail was observed in all individuals. The treatment with 30 μM JAS for 7 hours resulted in increased labelling of actin with a strong fluorescence signal, revealing its higher accumulation in longitudinal bands corresponding to the localisation of subpellicular filaments (Fig 9C). Nevertheless, the counterstaining with phalloidin did not show increased actin polymerisation in this area; the strong F-actin labelling of the parasite had diffuse character. The treatment with 10 μM cytochalasin D for 9 hours caused more homogenous labelling of actin with a medium intensity dispersed within the parasite cytoplasm, but only slightly decreased staining of F-actin (Fig 9D). The very strong labelling of myosin was restricted to the PS and host tissue (Fig 9E and 9H). Spectrin appeared to be dispersed in low concentrations in the parasite cytoplasm and surrounding host tissue (medium signal), while the PS, especially in the tail region, stained with a strong intensity (Fig 9F). Immunolabelling with an anti-α-tubulin antibody, used for visualisation of subpellicular microtubules and related structures, was repeatedly very strongly positive for the brush border of the host intestinal epithelium densely covered by microcilia. Both the parasite and the PS unexpectedly (formaldehyde is known to not satisfactory preserve microtubules) exhibited medium to strong labelling in PFA-fixed samples that were processed immediately after fixation (Fig 9G), while only weak to medium labelling was observed in those fixed in methanol (Fig 9H and 9I). Though more diffuse, the labelling of the same intensity was still noticeable at the parasite periphery after treatment with 10 μM oryzalin for 7 hours (Fig 9J). After incubation in 30 μM oryzalin for 3 hours, the peripheral labelling became weak to very weak in methanol-fixed samples, and putatively unpolymerised α-tubulin seemed to be more dispersed throughout the cytoplasm (Fig 9K and 9L). The very strong labelling of myosin in methanol-fixed samples was restricted to the periphery of PS and independent of oryzalin treatment (Fig 9H, 9J and 9K). High doses of oryzalin induced more frequent detachment of *E*. *duboscqi*, along with its sac, from the host tissue. To confirm that the modified microtubules were the reason for parasite detachment but not the redistribution of F-actin, the correct fixation for phalloidin staining was essential to retain the quaternary protein structure of F-actin (methanol destroys its native conformation and is not suitable for F-actin staining with phalloidin). Hence, control PFA fixation was performed for the co-localisation F-actin and α-tubulin. Parasites treated either with 10 μM or 30 μM oryzalin and subsequently fixed in PFA exhibited no changes in the F-actin distribution (Fig 9M and 9N), except for, when compared to the rest of PS, stronger labelling of the tail (Fig 9N). In contrast to methanol-fixed samples, the labelling with anti-α-tubulin antibody was almost undetectable in parasites and less conspicuous in the host brush border (Fig 9M–9O).

## Discussion

This study confirmed the epicellular localisation of the protococcidian *Eleutheroschizon duboscqi* on the gut epithelium of the polychaete *Scoloplos armiger*, as described in the original studies [10,11]. We use the term ‘parasitophorous sac’ to underline the peculiar localisation of the developmental stages of *E*. *duboscqi* in the host-derived two-membrane structure. Parasitophorous sac is the preferable term introduced for the first time by Paperna and Vilenkin [16] for the host-derived structure enveloping cryptosporidia. We believe that the term ‘parasitophorous vacuole’ to describe the location of epicellular organisms like *Cryptosporidium* and *Eleutheroschizon* is misleading, because it refers solely to a vacuolar space bordered by a membrane [5,17]. The parasitophorous sac (PS) is an epicellular structure (niche) enveloping the entire parasite composed of two continuous host plasma membranes on the outer and inner sides, enclosing a thin layer of host cell cytoplasm. In addition, a dense band of microfilaments separates the unmodified and modified parts of the host cell ([5], this manuscript).

### Attachment strategy and host-parasite interactions in *E*. *duboscqi* compared to other epicellular apicomplexans

**Fig 10:**
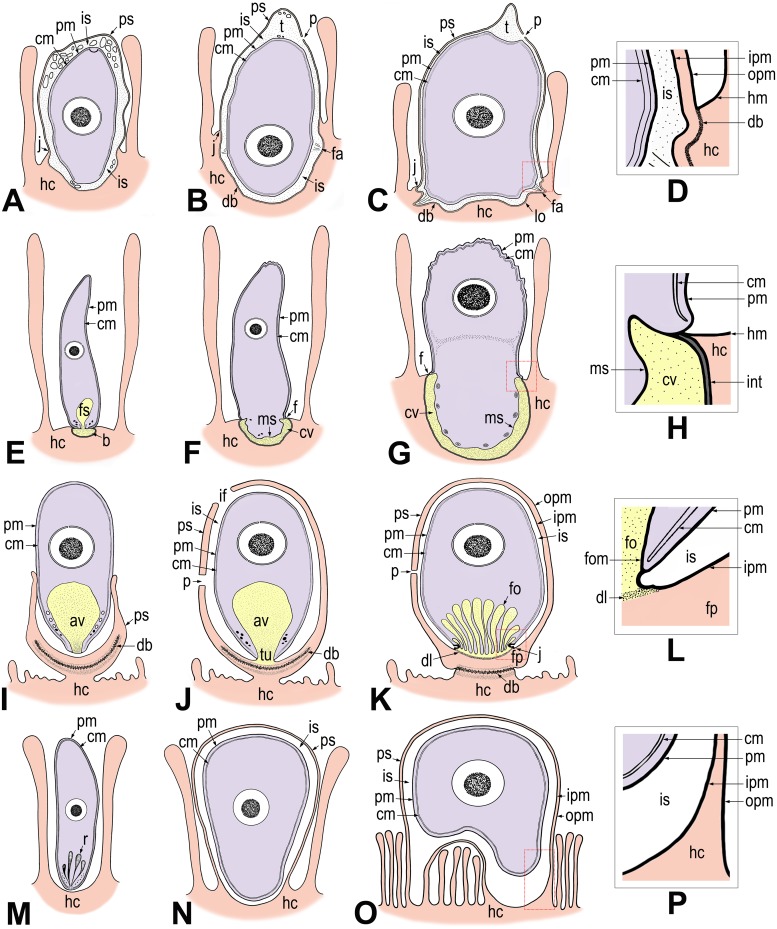
Schematic diagram of host-parasite interactions in *Eleutheroschizon duboscqi*, eugregarines, cryptosporidia, and epicellular eimeriids. The diagrams of *E*. *duboscqi*, gregarines and cryptosporidia are based on our personal observations enriched by published data. The diagram of eimeriids represents our interpretation and summary of published micrographs, where only maturing or mature stages were clearly shown [36–38,40,42– 44,64,65]. In this scheme, we refer to the host-derived envelope (described as a parasitophorous vacuole throughout literature) of eimeriids in epicellular location as a parasitophorous sac (PS) due to its organisation similar to that in cryptosporidia and *E*. *duboscqi*. Three colours are used to distinguish between the parasite (in purple), the host cell including its parts modified due to parasitisation (in pink) and the contact zone between the host and the parasite (in yellow) where the interrelationships of the two organisms become more intimate. In the case of host-parasite cellular interactions in *E*. *duboscqi* and epicellular eimeriids, the internal space between the parasite and PS remained colourless, even though we do not exclude the possibility that this region may serve as a transitional zone for intensive interactions between the host and its parasite. **A-D. *Eleutheroschizon duboscqi*. A.** Attached zoite transforming into a trophozoite stage, already completely enveloped by a PS. **B.** Maturing trophozoite with a forming ring of fascicles at the attachment site. The tail forms at the caudal part of the PS. **C.** Mature trophozoite with a prominent tail. Note the presence of attachment fascicles and lobes. **D.** Detailed view of the annular joint point (the cut-out is marked by a red square in C). **E-H. Eugregarines. E.** Sporozoite immediately after attachment to the host epithelial cell. **F.** Transformation of the sporozoite into a trophozoite stage. **G.** Early trophozoite with a well-developed epimerite. **H.** Detailed view of the membrane fusion site (the cut-out is marked by red square in G). The two cytomembranes end at the point of membrane fusion, where the osmiophilic ring is formed. **I-L. Cryptosporidia. I.** Attached zoite transforming into a trophozoite stage, partially enveloped by an incomplete PS. **J.** Young trophozoite almost completely enveloped by a PS. Note the tunnel connection between the interior of the anterior vacuole and the host cell cytoplasm that developed as the result of the Y-shaped membrane junction. **K.** Mature stage with a prominent filamentous projection at the base of the PS and with a fully developed feeder organelle, the lamellae of which formed from the anterior vacuole membrane. **L.** Detailed view of the Y-shaped membrane junction (the cut-out is marked by a red square in K). **M-P. Epicellular eimeriids. M.** Invading zoite. **N.** Trophozoite/meront stage enveloped by a PS with a single attachment area (monopodial form). **O.** Extension of the gamont stage above the microvillous region leading to an establishment of a new contact with the host cell apart from the primary attachment zone by penetration of the PS membrane to the base of fused microvilli (spider-like form). **P.** Detailed view of the attachment area (the cut-out is marked by a red square in O). *av*—anterior vacuole, *b—*epimeritic bud, *cm—*parasite cytomembranes, *cv—*epimeritic cortical vesicle, *db*—dense band (in cryptosporidia usually consisting of several layers), *f—*membrane fusion site, *dl—*dense line separating the feeder organelle from the filamentous projection of the PS, *fa*—attachment fascicle of filaments, *fo—*feeder organelle with membranous lamellae, *fom—*membrane limiting the lamellae of feeder organelle, *fp*—filamentous projection of the PS, *fs—*flask-shaped structure, *hc*—host cell, *hm*—host cell plasma membrane, *if—*incomplete fusion of PS, *int—*interface between the host cell and eugregarine epimerite, consisting of host cell plasma membrane, epimerite plasma membrane and a dense layer in between, *ipm*—inner membrane of the PS, *is—*internal space between the parasite and PS, *j*—annular joint point (Y-shaped membrane junction in cryptosporidia), *lo*—attachment lobe, *ms—*membrane-like structure limiting the cortical vesicle from the epimerite cytoplasm, *opm—*outer membrane of the PS, *p—*pore on the PS, *pm*—parasite plasma membrane, *ps*—parasitophorous sac, *r*—rhoptries, *t*—tail of the PS, *tu—*tunnel connection.

According to our analysis, *E*. *duboscqi* develops within the host-derived PS, resembling cryptosporidia [5,6,18,19,20]. Moreover, the parasite attaches to the host cell with the help of the complicated attachment site, by analogy with an invading gregarine [6,13,15,21–24] (Fig 10A–10L).

Gregarines are mostly intestinal epicellular parasites, equipped with a specialised attachment apparatus that might also serve as a feeding organelle (epimerite, mucron, modified protomerite) [13,15,21,22,25–29]. Archigregarines suck out the host cell cytoplasm using organelles of the apical complex [3,30,31]. Such a mechanism of feeding is called myzocytosis. On the contrary, many eugregarines seem to not feed through myzocytosis, except, probably, at their youngest developmental stages [32]. Their apical complex of organelles is reduced, and a new, more complicated, attachment apparatus forms (Fig 10G). This apparatus does not penetrate the host cell, but simply causes the invagination of the host plasmalemma (Fig 10E–10H) [6,15,22]. Cryptosporidia have a typical feeder organelle that attaches to the host cell and remains separated from the host cytoplasm by a dense line (Fig 10K and 10L) [5,6]. Similar to gregarines and cryptosporidia, *E*. *duboscqi* has a complicated attachment apparatus (fascicles and lobes in circles) (Fig 10C); and it remains unclear whether this apparatus is involved in the parasite feeding. Numerous pores are distributed along the entire pellicle of *E*. *duboscqi*, including the attachment site and some of them seem to be connected with vesicles and mitochondria. These pores may participate in parasite feeding. The apical complex of organelles is absent in *E*. *duboscqi* during the endogenous phase of its life cycle; and, apparently, feeding through myzocytosis is not typical for this parasite. Neither organelle similar to the flask-shaped structure described in the early stages of the eugregarines [6,13,22,23,32] nor the mucronal vacuole, characteristic of the archigregarine *Selenidium* [3,30], was detected in freshly attached *E*. *duboscqi* individuals. The flask-shaped structure observed in gregarines with an opening towards the apical pole (Fig 10E) initially appears electron-dense, but, with the formation of the cortical vesicle, it turns electron-lucent [6], suggesting that it might be a rhoptry emptying its enzymes. As rhoptry proteins are generally expected to be involved in the transformation of the host cell membrane into a parasitophorous vacuole (PV) [33], we cannot exclude the possibility that a similar structure may be present in *E*. *duboscqi* at a stage younger than the early trophozoite already enveloped by a PS.

Epicellular gregarines are usually not surrounded by any sac of host cell origin (Fig 10E–10H), except for the archigregarine *Ditrypanocystis* sp., which is enveloped by a multimembranous structure, originated from fused cilia of the host cell [24]. Cryptosporidia are enveloped by the PS, the inner membrane of which came from the plasmalemma presented on the host cell microvilli (Fig 10I–10L). In gregarines, as well as in cryptosporidia, the membrane fusion site is formed at the contact area between the parasite and host cell. In contrast, there is no connection between the host and *E*. *duboscqi* plasma membranes at the annular joint point. The internal space between the *E*. *duboscqi* plasmalemma and the inner membrane of the PS does not seem to be a part of the parasite. It rather resembles the space between the PV and intracellular coccidia located inside the host cell. As all early stages of *E*. *duboscqi* were seen to be completely contained within a PS, this host cell-derived envelope must develop much more rapidly than that documented in cryptosporidia, in which, during the invasion process, a tight-fitting membrane fold of the invaded host cell gradually rises up along the zoite, resulting in the formation of the PS [5,20]. The presence of a crystalloid body, a typical feature of sporozoites [26,34], in the stages attached and completely enveloped by the PS, confirms that the earliest observed stages were only slightly modified sporozoites, after their attachment to the host cell. Interestingly, the cortical vesicle in eugregarine *Didymophyes gigantea* was interpreted as a periparasitic space between the host and parasite, functioning as a PV [35]. At first glance, the gregarine cortical vesicle indeed resembles the internal space of the PV due to its translucent appearance with traces of an opaque or filamentous material [6,13,22,23]. In *Gregarina garnhami*, fine tubular structures pass through the cortical vesicle and attach to the epimerite-host cell interface [21]. We could speculate that the cortical vesicle is in fact an incomplete PV, restricted to the embedded apical region (epimerite) of the gregarine. It most likely develops from fused flat vesicles distributed in the epimerite periphery, originating from the parasite endoplasmic reticulum, and turns into a single large vesicle filled with microfilaments [23]. This vesicle is limited on its cytoplasmic face by a membranous structure, often discontinuous or multi-layered [6,15,21]. It retracts along with the epimerite during detachment of mature trophozoite from the host cell [15].

Epicellular localisation within the host tissue has also been described for certain eimeriid coccidia from fish (some *Eimeria* or former *Epieimeria*, some *Goussia*) [36–41] and reptiles (*Choleoeimeria*, *Acroeimeria*) [42,43]. According to multiple studies, they are localised at the enterocyte apical site among microvilli and covered by a double membrane envelope (the enterocyte and PV membranes), but a single PV membrane in contact areas (Fig 10M–10P). The arrangement of the PV membrane in contact zones is species specific based on various projections and undulations. No direct contact between the parasite and parasitised cell was observed [37,38,40,42–44]. During intracellular development of *Eimeria anguillae* merozoites, the PV with a parasite inside is expelled into the apical region of the parasitised cell, hereby taking an epicellular position [37]. In *Goussia pannonica* and *G*. *janae*, the parasites either attach to the host cell at a single contact area resulting from multiple fusions of microvilli (‘monopodial’ form) or are located above the microvillar zone, connected with the epithelium (occasionally to more than one enterocyte) through multiple thin projections in a spider-like arrangement [40,44]. The PV membrane of the spider-like projections have been described to be closely apposed to the enterocyte plasmalemma and to penetrate to the base of the villus where it contacts the host cytoplasm. The referenced micrographs, though not provided in satisfactory magnification, however, do not show any PV membrane penetrating into the host cytoplasm, which would indicate an intracellular (epicytoplasmic) position of the parasite. It rather suggests a deep invagination of the enterocyte membrane, at the connection with PV projections, i.e. epicellular localisation. Representatives of *Acroeimeria* are also considered to be epicellular parasites developing within a PV bulging above the epithelium surface [43]. From there, despite intracellular or epicellular initial stages of the development, some eimeriid coccidia of poikilothermic animals localise at the host cell apical part and are surrounded by the host-derived two-membrane PV.

Cryptosporidia, *E*. *duboscqi* and gregarines represent heteropolar cells; i.e. they exhibit a high degree of cell polarity in that their anterior and posterior ends differ in shape, structure and function. Extracellular but attached parasites are usually of a heteropolar nature, while the intracellular ones are generally non-polar. Epicellular eimeriids seem to be non-polar as they do not have any attachment organelles and seem to take up nutrients exclusively via the PV [37]. Nevertheless, they create projections of the PV [37,38] equipped with pores; these projections enlarge the contact area with the host cell and resemble the *E*. *duboscqi* attachment lobes and fascicles. Similarly, the complicated attachment organelles in many eugregarines seem to significantly increase the absorptive surface [13,29,35]. Apparently, the epicellular localisation, independent of its origin (whether initial stages of the parasite development are extracellular or intracellular), leads to the occurrence of cell polarity. All the above-mentioned apicomplexans form a specialised host-parasite interface, reflecting analogous modes of adaptation for survival and development in a similar host environment (i.e. the gastrointestinal epithelial brush border) [45] (Fig 10A–10P). It seems that, once attached to a proper host cell, all these parasites stimulate additional growth and subsequent fusion of host microvilli as well as further modifications to the host plasmalemma, leading to PS/PV formation. They tend to create a host-derived envelope around themselves and develop in its cavity so as to be separated from the host cytoplasm/environment. In cryptosporidia, the PS is not complete as they are directly connected to the host cell via a feeder organelle (i.e. they form the so-called Y-shaped junction between the host and parasite membranes), while in eimeriids and *E*. *duboscqi*, the inner membrane of this envelope contains the entire parasite. The mode of connection with the host cell remains inconclusive in the earliest developmental stages of *E*. *duboscqi*. Most likely it never invades the host cytoplasm, but attaches to the apical site of the host cell for a short time (until the formation of the PS).

It has been suggested that eimeriid epicellular development might be considered as a more primitive form of host-parasite association [42]. Some studies showed, however, that parasites developing epicellularly seem to have a less negative effect on the host epithelium than intracellular ones [46]. In general, it is more advantageous for the parasite to maintain its host in acceptable fitness; from this perspective, the evolution of this attachment strategy could be more progressive. While the most serious pathological changes in *E*. *anguillae* are induced by intracellular stages and vary depending on the intensity of parasitisation, the epicellular development causes alterations to the host epithelium, such as local swellings or a reduction in the number of microvilli [37]. Similar changes are reported in cryptosporidiosis, where the destiny of the host depends on its health status and immunocompetence [47,48]. In *E*. *anguillae*, the host epithelium, discharged from parasites, has lesions on the intestinal surface resembling circular holes [37]. Cryptosporidia and *E*. *duboscqi* leave only shallow craters with PS remains at the epithelial surface after their detachment [5]. Similarly, detached gregarine trophozoites leave flat holes in the epithelium lacking microvilli [15]. Microvilli are essential for digestion and nutrient absorption and their destruction might lead to host malnutrition with consequent weakening or even death. Although epicellular parasites destroy individual cells, the overall damage to epithelium in mild infections is negligible and often easily repaired thanks to its continual regeneration.

### Host actin distribution and architecture of *E*. *duboscqi* parasitophorous sac in comparison to cryptosporidia

Phalloidin staining, along with the application of drugs that influence the polymerisation of actin, confirmed the presence of actin filaments in host tissue and their increased accumulation in the PS surrounding *E*. *duboscqi*. Moreover, the higher doses of cytochalasin D, required for destroying actin filaments in the PS wall, suggest that the polymerised form of actin is more stable in the host-derived PS than in surrounding host tissue. This accumulation of host actin filaments might be induced by invading *E*. *duboscqi*, similarly to cryptosporidia, which induce the rearrangement and accumulation of actin and actin-binding proteins to their attachment site during invasion [49,50–52]. Cryptosporidia, however, show a low amount of F-actin in their PS [53]. Furthermore, the wall of the *E*. *duboscqi* PS exhibited a high accumulation of myosin. In cryptosporidia, the motor activity of host myosin seems to play an important role, putatively in association with microvilli extensions, in the formation of a parasite niche [49,52]. This theory is supported by a model for the protrusion of membranous structures, illustrating the significant involvement of myosin in the movement of actin filaments toward the apical surface of membrane extensions [54]. The involvement of host microcilia in the formation of the *E*. *duboscqi* PS is indicated by the presence of α-tubulin restricted to the PS wall, especially in the caudal region with the tail. The tubulin seemed to be in polymerised form, as incubation in oryzalin resulted in the vanishing of fluorescence as well as frequent detachment of parasites with their sacs from epithelium observed *in vitro*.

In contrast to intracellular coccidia, evolutionary selection presumably favoured the unique epicellular niche for cryptosporidia to more effectively evade the host immune response, though as a consequence, the attached parasite became dependent upon its connection with the host cell for nutrient acquisition. In *E*. *duboscqi*, the strategy could be similar. Host actin polymerisation and subsequent membrane protrusion are considered to be important for the establishment of a productive infection site in cryptosporidia [52], in which induced membrane extensions encapsulate the parasite and form the PS, with a dense band in the host cytoplasm located just beneath the attachment zone [5,6,20]. This band consists of electron-dense microfibrils interwoven perpendicularly [55], with an adjacent filamentous network of polymerised actin [52]. The dense band underlining the base of *E*. *duboscqi* is much thinner and closely apposed to the PS inner membrane. CLSM confirmed an increased accumulation of F-actin at the attachment site. In cryptosporidia, the actin reorganisation and formation of dense bands supported by the actin plaque were shown to be intimately involved in parasite anchoring and retention at the host cell apical surface [5,49,56]. An explanation for such a cytoskeletal rearrangement in parasitised cells is that this process results in the formation of a network for vesicle trafficking, facilitating the movement of nutrients between the host cell and the PS [49]. This hypothesis is in agreement with our observations on the accumulation of tiny filaments located within host cytoplasm surrounding the invaginations of PS membrane in the area of *E*. *duboscqi* attachment fascicles. These fascicles develop during *E*. *duboscqi* trophozoite maturation and seem to anchor the growing PS with the parasite to the host cell, while host filaments overlapping them could strengthen this fixation and prevent mechanical detachment.

Both parasites, cryptosporidia and *E*. *duboscqi*, regularly detach (most likely due to damage) along with their sacs from the unmodified part of the host cell. While the detachment of cryptosporidia takes place in the area of a dense band [5], individuals of *E*. *duboscqi* tear away from their sacs at the base, thereby exposing their naked basal region (i.e. covered by the parasite pellicle only) and leaving the intact inner membrane of the PS at the place of previous attachment. It seems that *E*. *duboscqi* induces only moderate alterations of the host cell in contrast to cryptosporidia, in which, along with actin remodelling, remarkable alteration of host membrane organisation has been documented [57]. Cytoskeletal remodelling of the host cell induced by cryptosporidia can be noted as microvillous hypertrophy, i.e. elongation and protrusion of host cell microvilli surrounding the parasite [49]. The persistence of long microvilli clustered at the attachment site suggests an active manipulation of the host membrane structure by the parasite. The microvilli associated with the cryptosporidian PS were particularly thick and contained dense bundles of F-actin. In *E*. *duboscqi*, we did not observe any significant extension of the adjacent host cell microvilli; only a few microcilia were occasionally attached to the PS surface (Fig 4A and 4K) and no obvious pathological changes were seen in the parasitised area. Despite a similar strategy of PS formation and architecture with cryptosporidia, epicellular stages of *E*. *duboscqi* seem to have less of a pathological effect on host tissue.

The function of the tail of the *E*. *duboscqi* PS remains unknown. Staining with Evans blue showed that the tail is protein-rich, confirmed by the SEM observations of the presence of a fibrous substance at the caudal region of parasites with a ruptured PS. Numerous pores along with a dense matrix, intensively stained by RR, in their vicinity indicate the role of the tail in the transportation of mucus and/or other parasite metabolites outside. Despite the presence of long filaments, the F-actin labelling in the tail turned out to be less distinct than the rest of the PS, but surprisingly, the incubation with oryzalin resulted in much stronger F-actin labelling in the tail in contrast to the proximal part of the PS. Tails of different sizes in maturing trophozoites may be a result of extra growth of the PS. Spectrin accumulation seemed to increase towards the PS caudal area with a tail, suggesting that plasma membrane proteins are accumulated in this zone. So, hypothetically, the fibrous substance could be a stock of membrane material needed for the PS development during the parasite maturation and growth. It remains unclear as to whether the ruptured PS observed in our preparations is a consequence of damage due to material processing or it represents a natural process in the life cycle allowing *E*. *duboscqi* gamonts to leave the host cell. It could be also a result of incomplete fusion of the PS during early parasite development.

### Cell cortex and cytoskeleton of *E*. *duboscqi*

In the course of transformation of *E*. *duboscqi* trophozoites into gamonts, a thick glycocalyx layer appears on the external surface of the parasite plasmalemma. Similar fibrillar coat, most likely of glycocalyx nature, has been documented covering the entire parasite in *Acroeimeria pintoi* [43]. In *E*. *duboscqi*, it forms earlier than the subpellicular filaments and seems to be essential for the attached stages. The most important role of glycocalyx might be a mechanical defence against potential fusion of the PS with the parasite surface. Importantly, the short and long attachment filaments that arise from the pellicle are anchored in the IMC and extend through the glycocalyx. They evidently represent a modified form of the glycocalyx.

The pellicle of *E*. *duboscqi* also appears unique in that it seems to re-build or reorganise during the parasite development. Vesicles observed under the plasma membrane in the area of discontinuous or absent cytomembranes of some gamonts indicate a process of membrane insertion into the cortex, most likely needed for pellicle completion during parasite growth. The barely distinguishable pellicle of early trophozoites provides further support for the hypothesis of its repetitive reorganisation. It is not clear how the parasite undergoes fertilisation and if its life cycle comprises additional free stages, but *in vitro* we often observed detachment of *E*. *duboscqi* along with PS, and documented detached parasites enveloped by the PS under electron microscope. All this suggests the participation of the PS in parasite protection from the surrounding environment even after detachment from the host tissue, probably as a consequence of pellicle reorganisation during *E*. *duboscqi* development.

The cytoskeleton of this parasite comprises subpellicular microtubules, but only during early development. While the posterior ring of *Toxoplasma gondii* does not connect with subpellicular microtubules [58], in *E*. *duboscqi* it does, similar to the ‘posterior polar ring’ in tachyzoites of *Besnoitia besnoiti* [59] or the so-called ‘proximal polar ring’ in *Plasmodium* sporozoites [60]. Usually, these structures are only recognised as pellicular (IMC) thickening. Although the microtubules disappeared during *E*. *duboscqi* trophozoite maturation and were not present in gamonts, we obtained positive α-tubulin labelling of the parasite surface and cytoplasm, suggesting the preservation of tubulin either in its non-polymerised form or its presence in other tubulin-rich structures. The second speculation seems to be more likely, because incubation in oryzalin resulted in the disappearance of labelling from the parasite periphery, and the putatively unpolymerised α-tubulin seemed to be more dispersed throughout the cytoplasm. Apicomplexan microtubules are selectively susceptible to drug-induced disruption; after prolonged treatment in 2.5 μM oryzalin all tubulin is usually unpolymerised and dispersed [61]. Oryzalin prevents the formation of microtubules in *T*. *gondii* daughter cells, but has a more moderate effect on existing microtubules in the mother cell [62,63]. This is in agreement with our results on *E*. *duboscqi*, where high drug doses must be applied for prolonged period to observe a dispersed character of tubulin staining. Of special interest are also the subpellicular bands of longitudinally oriented filaments, located beneath the parasite IMC, that form during the trophozoite maturation. Positive labelling of the parasite surface for actin/F-actin and the effect of cytoskeletal drugs on its staining suggest that these filaments are actin-rich. Considering the lack of subpellicular microtubules in mature stages, these bands could play the role of the parasite cytoskeleton.

## Conclusions

The endogenous stages of *Eleutheroschizon duboscqi* life cycle exclusively comprise trophozoites and gamonts. They develop in the intestine of *Scoloplos armiger* being attached to the host cell in an epicellular position and covered by a host-derived parasitophorous sac. Attached parasites share features of cryptosporidia and gregarines, i.e. they conspicuously resemble a maturing trophozoite of epicellular eugregarines with morphologically pronounced attachment apparatus, but are contained within a PS similar to that in cryptosporidia. However, *E*. *duboscqi* parasites have no intimate contact with the enterocyte membrane. The parasite pellicle seems to reorganise repeatedly during development. Detached parasites are enveloped by a PS covering their distal area above the attachment site, hereby suggesting that after detachment from the host tissue they preserve this envelope of host origin, providing them ongoing protection in a potentially unfriendly surrounding environment.

## References

[1] Adl SM, Simpson AG, Lane CE, Lukes J, Bass D, Bowser SS, et al The revised classification of eukaryotes. J Eukaryot Microbiol. 2012; 59: 429–493. 10.1111/j.1550-7408.2012.00644.x 23020233 PMC3483872

[2] Cox FEG. The evolutionary expansion of the Sporozoa. Int J Parasitol. 1994; 24: 1301–1316. 7729983 10.1016/0020-7519(94)90197-x

[3] Simdyanov TG, Kuvardina ON. Fine structure and putative feeding mechanism of the archigregarine *Selenidium orientale* (Apicomplexa: Gregarinomorpha). Eur J Protistol. 2007; 43: 17–25. 17126539 10.1016/j.ejop.2006.09.003

[4] Leander BS. Marine gregarines: evolutionary prelude to the apicomplexan radiation? Trends Parasitol. 2008; 24: 60–67. 10.1016/j.pt.2007.11.005 18226585

[5] Valigurova A, Jirku M, Koudela B, Gelnar M, Modry D, Slapeta J. Cryptosporidia: Epicellular parasites embraced by the host cell membrane. Int J Parasitol. 2008; 38: 913–922. 18158154 10.1016/j.ijpara.2007.11.003

[6] Valigurova A, Hofmannova L, Koudela B, Vavra J. An ultrastructural comparison of the attachment sites between *Gregarina steini* and *Cryptosporidium muris* . J Eukaryot Microbiol. 2007; 54: 495–510. 18070327 10.1111/j.1550-7408.2007.00291.x

[7] Barta JR, Thompson RCA. What is *Cryptosporidium*? Reappraising its biology and phylogenetic affinities. Trends Parasitol. 2006; 22: 463–468. 16904941 10.1016/j.pt.2006.08.001

[8] Carreno RA, Martin DS, Barta JR. *Cryptosporidium* is more closely related to the gregarines than to coccidia as shown by phylogenetic analysis of apicomplexan parasites inferred using small-subunit ribosomal RNA gene sequences. Parasitol Res. 1999; 85: 899–904. 10540950 10.1007/s004360050655

[9] Perkins FO, Barta JR, Clopton RE, Peirce MA, Upton SJ. Phylum Apicomplexa Levine, 1970 In: Lee JJ, Leedale GF, Bradbury P, editors. An illustrated guide to the Protozoa. 2nd ed. Society of Protozoologists, Lawrence, Kansas, USA; 2000 pp. 190–369.

[10] Chatton E, Villeneuve F. Le cycle évolutif del'*Eleutheroschizon duboscqui* Brasil. Preuve expérimentale de l'absence de schizogonie chez cette forme et chez la *Siedleckia caulleryi* Ch. et Vill. CR Acad Sci. 1936; 203: 833–836.

[11] Brasil L. *Eleutheroschizon duboscqi*, sporozoaire nouveau parasite de *Scoloplos armiger* O.F. Müller. Arch zool exp gen. 1906; 4: 17–22.

[12] Awerinzew S. Izsledovaniya nad’ paraziticheskimi prosteishimi I-VII. [Studies on parasitic protozoa I-VII. In Russian, German summary]. Trudy Imp S Petersburg Obsch Estestvoisp Vypusk 2: Otd Zool i Fiziol. 1908; 38: 1–139.

[13] Valigurova A. Sophisticated adaptations of *Gregarina cuneata* (Apicomplexa) feeding stages for epicellular parasitism. PLoS ONE. 2012; 7: e42606 10.1371/journal.pone.0042606 22900033 PMC3416826

[14] Sokolova YY, Paskerova GG, Rotari YM, Nassonova ES, Smirnov AV. Description of *Metchnikovella spiralis* sp. n. (Microsporidia: Metchnikovellidae), with notes on the ultrastructure of metchnikovellids. Parasitology. 2014; 141: 1108–1122. 10.1017/S0031182014000420 24813231

[15] Valigurova A, Michalkova V, Koudela B. Eugregarine trophozoite detachment from the host epithelium via epimerite retraction: Fiction or fact? Int J Parasitol. 2009; 39: 1235–1242. 10.1016/j.ijpara.2009.04.009 19460380

[16] Paperna I, Vilenkin M. Cryptosporidiosis in the gourami *Trichogaster leeri*: description of a new species and a proposal for a new genus, *Piscicryptosporidium*, for species infecting fish. Dis Aquat Organ. 1996; 27: 95–101.

[17] Scholtyseck E. Fine structure of parasitic protozoa: an atlas of micrographs, drawings and diagrams. Berlin: Springer-Verlag; 1979.

[18] Huang BQ, Chen XM, LaRusso NF. *Cryptosporidium parvum* attachment to and internalization by human biliary epithelia in vitro: A morphologic study. J Parasitol. 2004; 90: 212–221. 15165040 10.1645/GE-3204

[19] Umemiya R, Fukuda M, Fujisaki K, Matsui T. Electron microscopic observation of the invasion process of *Cryptosporidium parvum* in severe combined immunodeficiency mice. J Parasitol. 2005; 91: 1034–1039. 16419745 10.1645/GE-508R.1

[20] Lumb R, Smith K, Odonoghue PJ, Lanser JA. Ultrastructure of the attachment of *Cryptosporidium* sporozoites to tissue-culture cells. Parasitol Res. 1988; 74: 531–536. 3194366 10.1007/BF00531630

[21] Valigurova A, Koudela B. Morphological analysis of the cellular, interactions between the eugregarine *Gregarina garnhami* (Apicomplexa) and the epithelium of its host, the desert locust *Schistocerca gregaria* . Eur J Protistol. 2008; 44: 197–207. 10.1016/j.ejop.2007.11.006 18304787

[22] Valigurova A, Koudela B. Fine structure of trophozoites of the gregarine *Leidyana ephestiae* (Apicomplexa: Eugregarinida) parasitic in *Ephestia kuehniella* larvae (Lepidoptera). Eur J Protistol. 2005; 41: 209–218.

[23] Tronchin G, Schrevel J. Chronologie des modifications ultrastructurales au cours de la croissance de *Gregarina blaberae* . J Protozool. 1977; 24: 67–82. 405485 10.1111/j.1550-7408.1977.tb05282.x

[24] Butaeva F, Paskerova G, Entzeroth R. *Ditrypanocystis* sp. (Apicomplexa, Gregarinia, Selenidiidae): the mode of survival in the gut of *Enchytraeus albidus* (Annelida, Oligochaeta, Enchytraeidae) is close to that of the coccidian genus *Cryptosporidium* . Tsitologiia. 2006; 48: 695–704. 17147263

[25] Schrevel J, Vivier E. Étude de l’ultrastructure et du role de la région antérieure (mucron et épimérite) de grégarines parasites d’annélides polychètes. Protistologica. 1966; 2: 17–28.

[26] Desportes I. Ultrastructure et développement des grégarines du genre *Stylocephalus* . Ann Sci Nat Zool Paris. 1969; 12: 31–96.

[27] Schrevel J, Goldstein S, Kuriyama R, Prensier G, Vavra J. Biology of gregarines and their host-parasite interactions In: Desportes I, Schrevel J, editors. The gregarines: the early branching Apicomplexa: BRILL, NL; 2013 pp. 26–196.

[28] Baudoin J. The ultrastructure of the anterior region in the gregarine *Ancyrophora puytoraci* Protistologica. 1969; 5: 431–439.

[29] MacMillan WG. Gregarine attachment organelles—structure and permeability of an interspecific cell junction. Parasitology. 1973; 66: 207–214.

[30] Schrevel J. L'ultrastructure de la région antérieure de la grégarine *Selenidium* et son intérêt pour l'étude de la nutrition chez les sporozoaires. J Microsc. 1968; 7: 391–410.

[31] Schrevel J. Observations biologiques et ultrastructurales sur les Selenidiidae et leurs consequences sur la systematique des Gregarinomorphes. J Protozool 1971; 18: 448–470.

[32] Sheffield HG, Garnham PC, Shiroishi T. The fine structure of the sporozoite of *Lankesteria culicis* . J Protozool. 1971; 18: 98–105. 4993836 10.1111/j.1550-7408.1971.tb03289.x

[33] Dubremetz JF, Garcia-Reguet N, Conseil V, Fourmaux MN. Apical organelles and host-cell invasion by Apicomplexa. Int J Parasitol. 1998; 28: 1007–1013. 9724870 10.1016/s0020-7519(98)00076-9

[34] Roberts WL, Mahrt JL, Hammond DM. The fine structure of the sporozoites of *Isospora canis* . Z Parasitenkd. 1972; 40: 183–194. 4346237 10.1007/BF00329620

[35] Hildebrand HF. Electron-microscopic investigation on evolution stages of trophozoite of *Didymophyes gigantea* (Sporozoa, Gregarinida). 1. Fine structure of protomerite and epimerite and relationship between host and parasite. Z Parasitenkd. 1976; 49: 193–215. 824879 10.1007/BF00380590

[36] Lukes J. A coccidian (Apicomplexa: Eimeriidae) with extracytoplasmally located stages in the kidney tubules of golden carp (*Carassius auratus gibelio* L.) (Cyprinidae). Folia Parasitol. 1993; 40: 1–7. 8325562

[37] Benajiba MH, Marques A, Lom J, Bouix G. Ultrastructure and sporogony of *Eimeria* (syn. *Epieimeria*) *anguillae* (Apicomplexa) in the eel (*Anguilla anguilla*). J Eukaryot Microbiol. 1994; 41: 215–222.

[38] Molnar K, Baska F. Light and electron microscopic studies on *Epieimeria anguillae* (Léger & Hollande, 1922), a coccidium parasitizing the European eel, *Anguilla anguilla* L. J Fish Dis. 1986; 9: 99–110.

[39] Dyková I, Lom J. Fish coccidia: critical notes on life cycles, classification and pathogenicity. J Fish Dis. 1981; 4: 487–505.

[40] Lukes J. Life cycle of *Goussia pannonica* (Molnar, 1989) (Apicomplexa, Eimeriorina), an extracytoplasmic coccidium from the white bream *Blicca bjoerkna* . J Protozool. 1992; 39: 484–494.

[41] Jirku M, Modry D, Slapeta JR, Koudela B, Lukes J. The phylogeny of *Goussia* and *Choleoeimeria* (Apicomplexa; Eimeriorina) and the evolution of excystation structures in coccidia. Protist. 2002; 153: 379–390. 12627867 10.1078/14344610260450118

[42] Paperna I, Landsberg JH. Description and taxonomic discussion of eimerian coccidia from African and Levantine geckoes. S A J Zool. 1989; 24: 345–355.

[43] Paperna L, Lainson R. Fine structure of the epicytoplasmic eimerid coccidium *Acroeimeria pintoi* Lainson & Paperna, 1999, a gut parasite of the lizard *Ameiva ameiva* in north Brazil. Parasite. 1999; 6: 359–364. 10633508 10.1051/parasite/1999064359

[44] Lukes J, Stary V. Ultrastructure of the life-cycle stages of *Goussia janae* (Apicomplexa, Eimeriidae), with X-ray microanalysis of accompanying precipitates. Can J Zool. 1992; 70: 2382–2397.

[45] Ortega-Pierres G, Caccio S, Fayer R, Mank TG, Smith HV, Thompson RCA. *Giardia* and *Cryptosporidium*: From molecules to disease. CABI Publishing; 2009.

[46] Eli A, Briyai OF, Abowei JFN. A Review of some parasite diseases of African fish gut lumen Protozoa, Coccidioses, *Cryptosporidium* infections, Haemoprotozoa, Haemosporidia. Res J Appl Sci Eng Technol. 2012; 4: 1438–1447.

[47] Melicherova J, Ilgova J, Kvac M, Sak B, Koudela B, Valigurova A. Life cycle of *Cryptosporidium muris* in two rodents with different responses to parasitization. Parasitology. 2014; 141: 287–303. 10.1017/S0031182013001637 24128742

[48] Bouzid M, Hunter PR, Chalmers RM, Tyler KM. *Cryptosporidium* pathogenicity and virulence. Clin Microbiol Rev. 2013; 26: 115–134. 10.1128/CMR.00076-12 23297262 PMC3553671

[49] Forney JR, DeWald DB, Yang SG, Speer CA, Healey MC. A role for host phosphoinositide 3-kinase and cytoskeletal remodeling during *Cryptosporidium parvum* infection. Infect Immun. 1999; 67: 844–852. 9916099 10.1128/iai.67.2.844-852.1999 PMC96395

[50] Elliott DA, Coleman DJ, Lane MA, May RC, Machesky LM, Clark DP. *Cryptosporidium parvum* infection requires host cell actin polymerization. Infect Immun. 2001; 69: 5940–5942. 11500478 10.1128/IAI.69.9.5940-5942.2001 PMC98718

[51] Hashim A, Mulcahy G, Bourke B, Clyne M. Interaction of *Cryptosporidium hominis* and *Cryptosporidium parvum* with primary human and bovine intestinal cells. Infect Immun. 2006; 74: 99–107. 16368962 10.1128/IAI.74.1.99-107.2006 PMC1346631

[52] O'Hara SP, Small AJ, Chen XM, LaRusso NF. Host cell actin remodeling in response to *Cryptosporidium* . Subcell Biochem. 2008; 47: 92–100. 18512344 10.1007/978-0-387-78267-6_7

[53] Bonnin A, Lapillonne A, Petrella T, Lopez J, Chaponnier C, Gabbiani G, et al Immunodetection of the microvillous cytoskeleton molecules villin and ezrin in the parasitophorous vacuole wall of *Cryptosporidium parvum* (Protozoa: Apicomplexa). Eur J Cell Biol. 1999; 78: 794–801. 10604656 10.1016/S0171-9335(99)80030-2

[54] Mitchison TJ, Cramer LP. Actin-based cell motility and cell locomotion. Cell. 1996; 84: 371–379. 8608590 10.1016/s0092-8674(00)81281-7

[55] Landsberg JH, Paperna I. Ultrastructural study of the coccidian *Cryptosporidium* sp. from stomachs of juvenile cichlid fish. Dis Aquat Organ. 1986; 2: 13–20.

[56] Elliott DA, Clark DP. *Cryptosporidium parvum* induces host cell actin accumulation at the host-parasite interface. Infect Immun. 2000; 68: 2315–2322. 10722635 10.1128/iai.68.4.2315-2322.2000 PMC97419

[57] Yoshikawa H, Iseki M. Freeze-fracture study of the site of attachment of *Cryptosporidium muris* in gastric glands. J Protozool. 1992; 39: 539–544. 1387896 10.1111/j.1550-7408.1992.tb04848.x

[58] Ferguson DJ, Sahoo N, Pinches RA, Bumstead JM, Tomley FM, Gubbels MJ. MORN1 has a conserved role in asexual and sexual development across the Apicomplexa. Eukaryot Cell. 2008; 7: 698–711. 10.1128/EC.00021-08 18310354 PMC2292627

[59] Reis Y, Cortes H, Viseu Melo L, Fazendeiro I, Leitao A, Soares H. Microtubule cytoskeleton behavior in the initial steps of host cell invasion by *Besnoitia besnoiti* . FEBS Letters. 2006; 580: 4673–4682. 16876796 10.1016/j.febslet.2006.07.050

[60] Kudryashev M, Lepper S, Stanway R, Bohn S, Baumeister W, Cyrklaff M, et al Positioning of large organelles by a membrane-associated cytoskeleton in *Plasmodium* sporozoites. Cell Microbiol. 2010; 12: 362–371. 10.1111/j.1462-5822.2009.01399.x 19863555

[61] Beck JR, Rodriguez-Fernandez IA, de Leon JC, Huynh MH, Carruthers VB, Morrissette NS, et al A novel family of *Toxoplasma* IMC proteins displays a hierarchical organization and functions in coordinating parasite division. PLoS Pathog. 2010; 6: e1001094 10.1371/journal.ppat.1001094 20844581 PMC2936552

[62] Morrissette NS, Mitra A, Sept D, Sibley LD. Dinitroanilines bind alpha-tubulin to disrupt microtubules. Mol Biol Cell. 2004; 15: 1960–1968. 14742718 10.1091/mbc.E03-07-0530 PMC379290

[63] Morrissette NS, Sibley LD. Disruption of microtubules uncouples budding and nuclear division in *Toxoplasma gondii* . J Cell Sci. 2002; 115: 1017–1025. 11870220 10.1242/jcs.115.5.1017

[64] Kim SH, Paperna I. Fine structure of epicytoplasmic stages of *Eimeria vanasi* from the gut of cichlid fish. Dis Aquat Organ. 1992; 12:191–197.

[65] Paperna I, Landsberg JH. Tubular formations extending from parasitophorous vacuoles in gut epithelial cells of cichlid fish infected by *Eimeria* (s. l.) *vanasi* . Dis Aquat Organ. 1987; 2: 239–242.

